# High-Throughput Combinatorial Analysis of the Spatiotemporal Dynamics of Nanoscale Lithium Metal Plating

**DOI:** 10.1021/acsnano.4c05001

**Published:** 2024-08-13

**Authors:** Daniel Martín-Yerga, Xiangdong Xu, Dimitrios Valavanis, Geoff West, Marc Walker, Patrick R. Unwin

**Affiliations:** † Department of Chemistry, University of Warwick, Coventry CV4 7AL, U.K.; ‡ Department of Chemistry, Nanoscience Center, University of Jyväskylä, Jyväskylä 40100, Finland; § Warwick Manufacturing Group, University of Warwick, Coventry CV4 7AL, U.K.; ∥ Department of Physics, University of Warwick, Coventry CV4 7AL, U.K.

**Keywords:** lithium-ion battery, lithium plating, nucleation and growth, scanning electrochemical cell microscopy, opto-electrochemistry, combinatorial electrochemistry

## Abstract

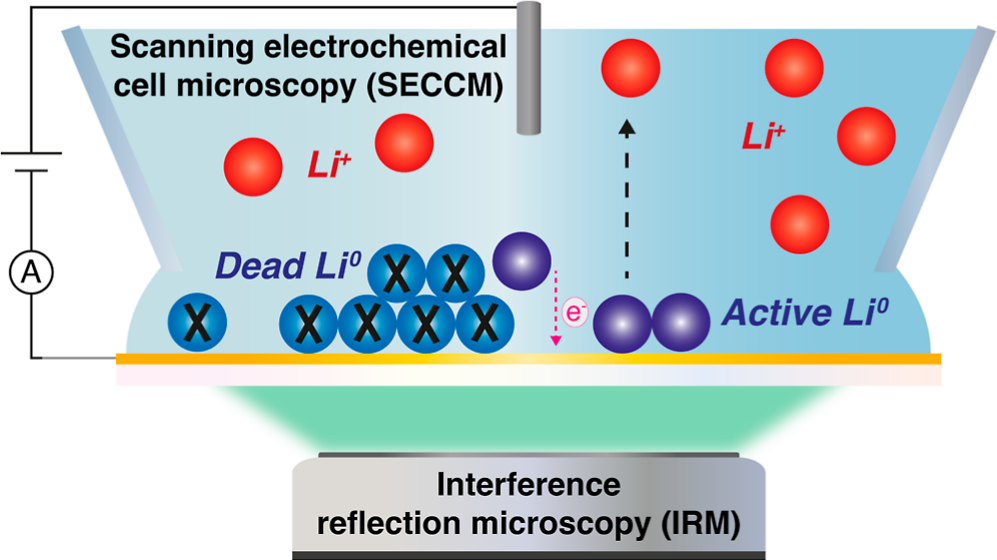

The development of Li metal batteries requires a detailed understanding of complex nucleation and growth processes during electrodeposition. *In situ* techniques offer a framework to study these phenomena by visualizing structural dynamics that can inform the design of uniform plating morphologies. Herein, we combine scanning electrochemical cell microscopy (SECCM) with *in situ* interference reflection microscopy (IRM) for a comprehensive investigation of Li nucleation and growth on lithiophilic thin-film gold electrodes. This multimicroscopy approach enables nanoscale spatiotemporal monitoring of Li plating and stripping, along with high-throughput capabilities for screening experimental conditions. We reveal the accumulation of inactive Li nanoparticles in specific electrode regions, yet these regions remain functional in subsequent plating cycles, suggesting that growth does not preferentially occur from particle tips. Optical-electrochemical correlations enabled nanoscale mapping of Coulombic Efficiency (*CE*), showing that regions prone to inactive Li accumulation require more cycles to achieve higher *CE*. We demonstrate that electrochemical nucleation time (*t*_nuc_) is a lagging indicator of nucleation and introduce an optical method to determine *t*_nuc_ at earlier stages with nanoscale resolution. Plating at higher current densities yielded smaller Li nanoparticles and increased areal density, and was not affected by heterogeneous topographical features, being potentially beneficial to achieve a more uniform plating at longer time scales. These results enhance the understanding of Li plating on lithiophilic surfaces and offer promising strategies for uniform nucleation and growth. Our multimicroscopy approach has broad applicability to study nanoscale metal plating and stripping phenomena, with relevance in the battery and electroplating fields.

## Introduction

The increasing need to electrify the economy, reduce greenhouse gas emissions and create a sustainable society requires the development of batteries with high energy density. Lithium (Li) metal is a promising candidate as an energy-dense negative electrode for future battery architectures,^1,2^ particularly when used in an anode-less configuration.^3^ This configuration optimizes the active material loading by directly plating the metal onto the current collector. However, Li metal batteries have shown relatively low performance, capacity losses and safety issues due to uncontrolled dendrite growth and accumulation of dead Li upon battery cycling.^4^ Mechanisms of dendrite formation are not yet well understood since they are determined by multiple factors such as applied current density, overpotentials, electrolyte composition and electrode structure.^5^ The space charge model predicts that dendrites form when the reaction becomes mass-transport controlled,^6^ which occurs at high current densities,^7^ while small heterogeneities on the electrode surface such as local protrusions promote preferential metal growth.^8^ Dendrites have also been observed at low current densities,^7^ which suggests that other phenomena can influence their formation, such as the local electric field,^9^ the solid–electrolyte interphase (SEI),^10−12^ and other factors that remain unclear. Dendritic formations have been observed even in solid-state batteries.^13^ Understanding Li nucleation and growth, and the associated dynamics, is critical for the development of high performing and dendrite-free Li metal batteries, which will significantly benefit future energy storage and utilization systems.

Several strategies^14^ based on particular electrolytes,^15^ operating conditions such as pulse charging,^16−18^ artificial SEI layers,^19^ and three-dimensional current collectors^20,21^ have been attempted to mitigate dendrite formation. One of the increasingly popular approaches is to use a lithiophilic metal as the current collector, which is capable of forming Li-M alloys, M being gold, zinc, tin, and others.^22−24^ These alloys act as nucleation centers, making Li growth more uniformly and reducing nucleation overpotentials. Gold (Au) has strong affinity for Li, forming a series of intermetallic alloys with different crystalline phases^25,26^ and displaying a complex electrochemical response.^27^ Au has only partial solubility in Li and after formation of the highest concentrated alloy (Li_15_Au_4_), Li deposition can also occur.^23^ The alloying phenomenon promotes the preferential deposition of Li metal on Au centers such as nanoparticles^28,29^ and surface nanopatterns,^30^ due to the low (or nonexistent) nucleation overpotential of Li on Li_*x*_Au_*y*_,^28^ and ultimately leads to a more uniform plating and improved cycling stability.

Li electrodeposition has also been found to be dependent on the morphology of Au seeds,^30^ and the growth of Li metal on Au can also be affected by the SEI layer formation and evolution.^31^ Continuous cycling can also cause degradation of Au films^32^ due to cracking^33^ and material pulverization, as a result of the volume expansion and contraction cycles.^34,35^ Lithiophilic materials are not as commonly studied as copper current collectors, and further research is needed to fully understand their behavior. This is particularly important for the early stages of Li metal deposition, involving nucleation and nucleus growth,^36,37^ which play a critical role in determining the ultimate structural properties of Li deposits.

*In situ* imaging techniques that allow for real-time visualization of Li deposition are essential to enhance our mechanistic understanding of this complex process.^38^ However, existing techniques present limitations in either the spatial or time resolution necessary for studying the early stages of deposition, or are low-throughput and thus limited to exploring a narrow set of conditions. For instance, conventional optical microscopy is compatible with operando metal deposition,^39^ but lacks the necessary resolution to detect Li deposits with submicrometer sizes due to the diffraction limit. Synchrotron-based X-ray techniques^40−42^ and magnetic resonance imaging^43^ have been used to visualize Li plating and stripping, but also lack spatial resolution. Surface plasmon resonance^44^ is highly sensitive and can detect thin Li deposits (consisting of a few atomic layers), but it has not yet been used for spatial imaging of Li plating. *In situ* electron^45,46^ and atomic force^47,48^ microscopies provide excellent spatial resolution for imaging but require particular conditions and time-consuming measurements, thereby limiting the experimental space that can be accessed. There is a clear need for further *in situ* experimental configurations that enable imaging of the initial stages of Li metal deposition to accelerate the understanding of Li deposition and stripping dynamics.

**Figure 1:**
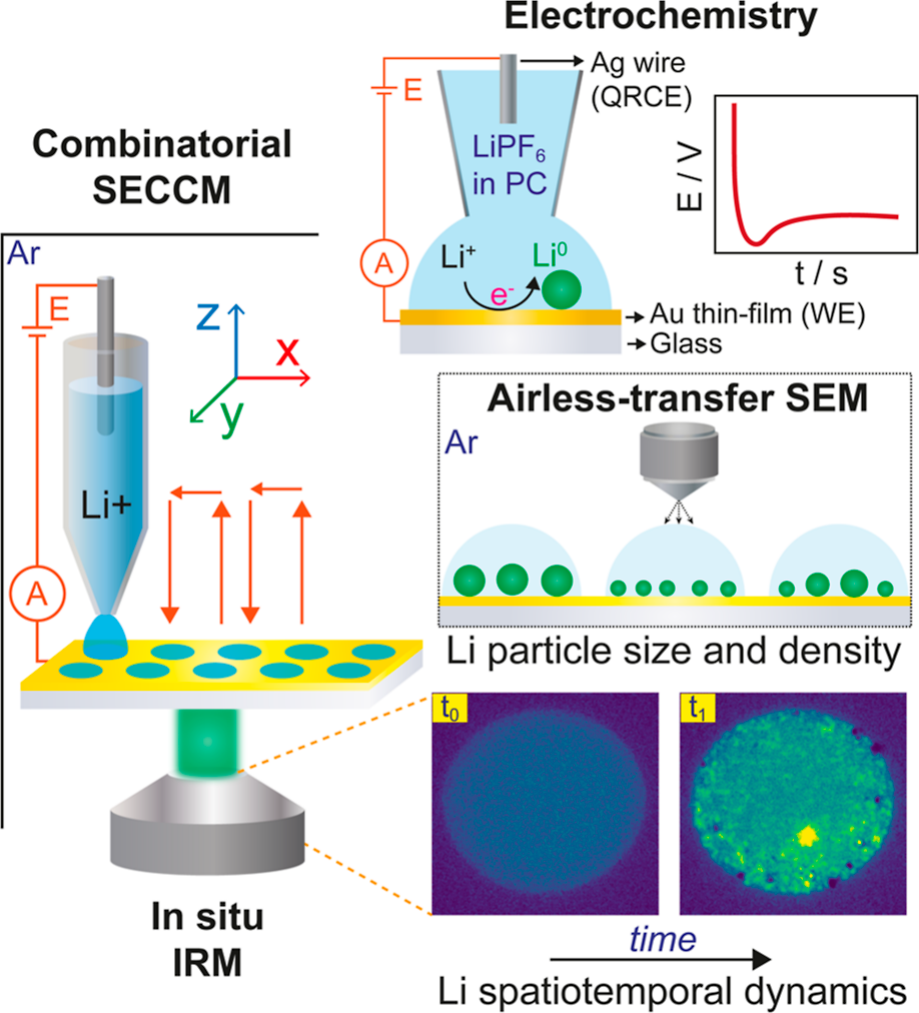
Schematic illustrating the combined *in situ* SECCM/IRM in an Ar-filled glovebox and *ex situ* airless-transfer SEM multimicroscopy approach, designed to correlate combinatorial electrochemical measurements with the spatiotemporal dynamics of Li plating and Li particle size and areal density. The SECCM system included an Au thin-film as working electrode (WE) and an Ag wire as quasi-reference and counter electrode (QRCE). Li plating and stripping was monitored on the WE.

Here, we present an *in situ* multimicroscopy approach, based on coupling scanning electrochemical cell microscopy with interference reflection microscopy (SECCM/IRM), to study the spatiotemporal dynamics of Li plating and stripping on lithiophilic thin-film Au electrodes, as illustrated in Figure 1. Our approach allows for visualization of Li plating with high spatial resolution (∼40 nm), fast time resolution (ca. 250 ms/frame herein, but faster possible), optical–electrochemical correlation, and high-throughput capabilities for combinatorial screening of plating conditions. Further correlation of these data with *ex situ* scanning electron microscopy (SEM) and secondary ion mass spectrometry (SIMS) provides additional information about Li deposits, such as particle size and areal density.

Pipette-based techniques, including SECCM and scanning ion conductance microscopy (SICM), are emerging as key tools for achieving a localized understanding of battery materials and interphases. SICM has enabled the monitoring of ion–concentration profiles and topography changes during charging/discharging.^49^ SECCM has provided insights on SEI formation and dynamics,^50−52^ nanoscale interfacial degradation,^53^ single-nanoparticle lithiation,^54−56^ and spatially resolved electrochemical imaging.^57^ SECCM/IRM has previously enabled the targeting of nanoparticles for smart localized measurements,^56^ and the visualization of single particle nucleation and dynamics^58−60^ by detecting optical features related to local changes in refraction index, and surpassing the diffraction limit constraint. Indeed, detection of nanoparticles as small as 10 nm has been previously reported.^61^ Our studies herein focus on the early stages of Li nucleation and growth, at a relatively low state of charge, where small (<100 nm) Li nanoparticles (LiNPs) are generally observed, depending on conditions. In SECCM, each measurement position can be tailored to a specific set of conditions, and this enabled us to perform a series of experiments such as dynamic plating and stripping, combinatorial plating under different current densities, and spatiotemporal monitoring of Li plating. Through this combinatorial strategy, we unveil major insights on Li electrodeposition such as the buildup of inactive Li upon cycling, and important factors controlling Li nucleation and growth such as current density, local structural features of the electrode, and local mass transport regimes. Our approach also provides opportunities to study electrode wetting effects by the droplet configuration, it is not limited to Li and can be broadly applied to reveal phenomena on metal nucleation and growth relevant to batteries (Na, K, Mg, Zn, etc.), and beyond, within the broad field of electrochemical plating.

## Results and Discussion

### Lithium Plating and Stripping Dynamics on Gold Thin-Film Electrodes

**Figure 2:**
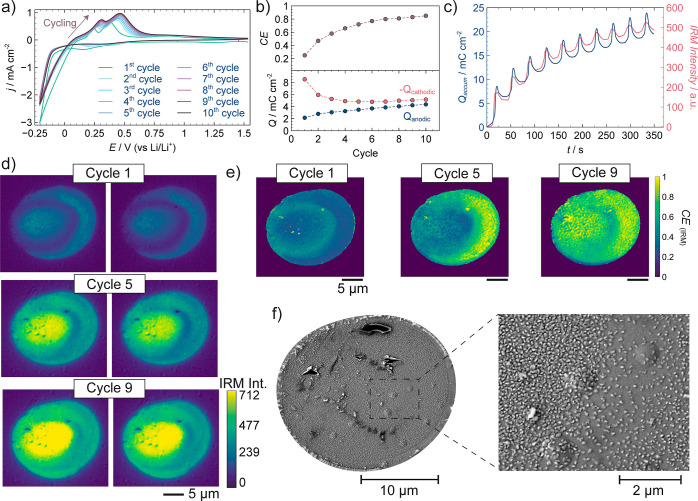
(a) CV (10 cycles) recorded by SECCM with a cutoff potential of −0.27 V vs Li/Li^+^. Measurements were conducted using a pipet with a diameter of ca. 10 μm filled with 50 mM LiPF_6_ in a PC solution. Scan rate was 100 mV s^–1^. (b) Evolution of Coulombic efficiency (*CE*) (top) and anodic and cathodic charges (bottom) as a function of cycle number. (c) Changes in accumulated charge (blue line) and IRM intensity (averaged across the SECCM landing/deposition area, red line) over the experimental duration. (d) IRM images captured after Li deposition (left) and after Li stripping (right) for the first, fifth and ninth voltammetric cycles. (e) Spatially resolved *CE*_(IRM)_ maps calculated for the first, fifth and ninth cycle. Note that a few local spots within these maps correspond to decreased IRM intensities resulting from very thick Li deposits, and as such, they are not considered in the discussion. (f) SEM image taken after the SECCM voltammetric experiment (following the 10th stripping cycle, ending at +1.47 V vs Li/Li^+^) with a magnified view (inset) of a portion of the SECCM footprint.

*In situ* SECCM/IRM was initially used to monitor potential-dependent Li metal plating and stripping dynamics on thin-film Au electrodes (see characterization in Figure S1) by cyclic voltammetry (CV). We used a pipet with a diameter of ca. 10 μm (Figure S2a), filled with a solution of 50 mM LiPF_6_ in propylene carbonate (PC), to record a set of 10 voltammetric cycles between +1.47 V and −0.27 V vs Li/Li^+^ at a scan rate of 100 mV s^–1^ (Figure 2a). The cathodic sweep of the first cycle revealed distinct processes and slightly higher current densities than subsequent cycles, attributed to the formation of the SEI layer on the Au surface.^34^ The most significant cathodic process was Li plating at an onset potential ca. −0.13 V vs Li/Li^+^. The three anodic processes with peak potentials +0.18, +0.32, and +0.82 V vs Li/Li^+^ are assigned to Li stripping and Li_*x*_Au_*y*_ dealloying. Recording a CV up to 0 V (Figure S3), where Li plating should be minimal or nonexistent, confirmed the alloy formation. The cathodic response under this cutoff potential remained intricate due to the SEI formation and Li_*x*_Au_*y*_ alloying with phase transformations. Indeed, initial metastable alloys such as Li_3_Au_2_ and Li_5_Au_3_ have been previously observed,^26^ which are transformed to a Li_15_Au_4_ phase with an increase of Li content.^23^ However, the anodic sweep only featured a single oxidation process ca. + 0.85 V vs Li/Li^+^ that coincides with the third anodic peak in the CV for a more negative cutoff potential (Figure 2a). This process is due to Li stripping (dealloying) from a Li_*x*_Au_*y*_ phase with low Li content such as Li_3_Au_2_ or LiAu_3_, which have been previously detected during the stripping process.^23,35^ We thus tentatively attribute the remaining two anodic processes to Li stripping from pure Li metal, likely the process at the less positive potential, and from a Li-richer Li_*x*_Au_*y*_ alloy such as Li_15_Au_4_ or Li_3_Au.^23,26^

The variation of cathodic (*Q*_c_) and anodic (*Q*_a_) charges and their ratio *Q*_a_/*Q*_c_ (i.e., Coulombic efficiency, *CE*) as a function of cycle number is presented in Figure 2b. This analysis tracks the reversibility of Li plating and stripping. *CE* increased from 0.25 in the first cycle to 0.85 by the 10th cycle, mainly due to a steep decrease of *Q*_c_ in the initial cycles, coupled with a gradual increase of *Q*_a_. Although the SEI formation (i.e., electrolyte reactivity) can partly explain this trend, clear losses of Li were observed during these initial cycles even though the reversibility still displayed an upward trend during the latter cycles. Indeed, the low *CE* has been found to be governed by the formation of unreacted metallic Li.^62^ Dead Li has been observed on Au electrodes by the formation of nanoparticles that become trapped in the SEI layer^34^ and thus electrically detached from the current collector.

We used the *in situ* recorded IRM movies to obtain further information about the dynamics of Li plating/stripping. Figure 2d shows selected IRM frames at specific times corresponding with the first, fifth and ninth cycles of Li deposition (left) and stripping (right). A larger number of frames are presented in Figure S4, and the full movie is shown in Movie S1. The IRM intensity (averaged across the SECCM landing/deposition area) increased during the plating cycles as it is sensitive to local refractive index changes at the electrode interface caused by the SEI formation,^63^ as well as Li metal deposition and Li_*x*_Au_*y*_ formation. After each stripping cycle, the IRM intensity decreased but never reached the same value as before the corresponding plating cycle (Figure 2c, red line). This suggests that there is accumulation of inactive Li on particular regions of the electrode (Figure 2d), predominantly LiNPs as confirmed by the strong correlation with colocated SEM imaging (vide infra). The IRM intensity not only reflects the local change in refractive index, but also the local thickness or mass of deposited material.^61^ Indeed, the reflectance change is generally proportional to the amount of deposited material when the thickness values are small compared to the wavelength,^61^ and for the specific case of spherical nanoparticles,^64^ according to eq 1
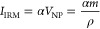
1where *I*_IRM_ is the IRM intensity, α is a correlation factor that depends on electrode thickness and optical noise,^61^*V*_NP_ is the volume of the nanoparticle, *m* is the mass of the nanoparticle, and ρ is the density of the material. The cumulative electrochemical charge reflected the same behavior as the IRM intensity with a strong correlation (Figure 2c, blue line), and from Faraday’s laws, the relationship between mass and charge, eq 2 is obtained
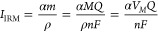
2where *Q* is the charge transferred, *M* is the molar mass of the material, *n* is the number of electrons transferred by the reaction, *F* is the Faraday constant, and *V*_M_ is the molar volume of material.

This analysis demonstrates IRM as an excellent technique not only to visualize, but also to semiquantify Li metal plating and stripping with nanoscale resolution and to monitor the buildup of inactive (dead) deposits, which is an ongoing challenge in this field.^62,65^ In this regard, a good correlation between *CE* and the ratio between the change in IRM intensity after stripping (*I*_a_) and after plating (*I*_c_) was also found (Figure S5). Consequently, IRM can further provide spatially resolved trends in *CE* during cycling, with simultaneous information about local regions where plating, stripping and accumulation of inactive Li are enhanced. The evident relationship between *I*_a_/*I*_c_ and *CE* ratio (above) can be used to reveal the spatially resolved *CE*, named *CE*_(IRM)_ herein, at different locations across the electrode surface, *pixel-by-pixel* (∼40 nm resolution), as shown in Figure 2e for the first, fifth, and ninth cycle, with histograms shown in Figure S6. Our analysis (full sequence of cycles in Figure S7) reveals that local regions of the electrode surface, where Li plating is promoted and inactive Li accumulates, exhibit a lower *CE*_(IRM)_ during initial cycles. However, as the number of cycles increases (around eight to ten cycles), a more homogeneous *CE*_(IRM)_ across the entire surface is observed. Despite this trend toward spatial homogeneity and *CE*_(IRM)_ values closer to 1, the absolute IRM intensity still shows certain regions that are rich in inactive Li. These findings suggest that electrode regions where inactive Li is formed do not necessarily become entirely inoperative during subsequent cycles. This phenomenon aligns with previous observations, such as Li metal deposits growing from the base rather than the tips,^66^ or deposition being possible on inactive metal due to the formation of an electric potential field in the electrolyte.^65^

After SECCM cycling, we collected SEM images from the SECCM footprint (Figure 2f) to examine the Li deposits on the electrode surface. The SEM images revealed many LiNPs with a mean diameter of 72 ± 26 nm (Figure S8) and an areal particle density of 5.3 × 10^9^ particles cm^–2^. These LiNPs are mostly inactive Li or at least Li that is not effectively removed from the surface, as the images were taken after the 10-cycle stripping (ending at a potential of +1.53 V vs Li/Li^+^), confirming the IRM findings about the local Li buildup. Indeed, there was also a good correlation between the local *in situ* IRM absolute intensity (last frame in Figure S4) and particle density from SEM. The center of the SECCM probed area contained a higher particle density (Figure 2f) associated with a higher IRM intensity demonstrating that IRM successfully detects the accumulation of even very small LiNPs (most of them well below 100 nm diameter). Note that in this case the pipet position above the wetted area was slightly off-center, as shown in Figure 2d–e, and that this is readily detectable by *in situ* IRM. We confirmed the presence of Li across the SECCM probed area by correlative SIMS Li imaging (Figure S9), acknowledging that SIMS also detects Li from Li_*x*_Au_*y*_ alloys and electrolyte salts that remain on the surface (samples were not rinsed to avoid damaging or altering the original structure), and the fact that many of the LiNPs were relatively small compared to the SIMS lateral resolution under these conditions (ca. 27 nm per pixel).

### High-Throughput Combinatorial Analysis of Lithium Plating

**Figure 3:**
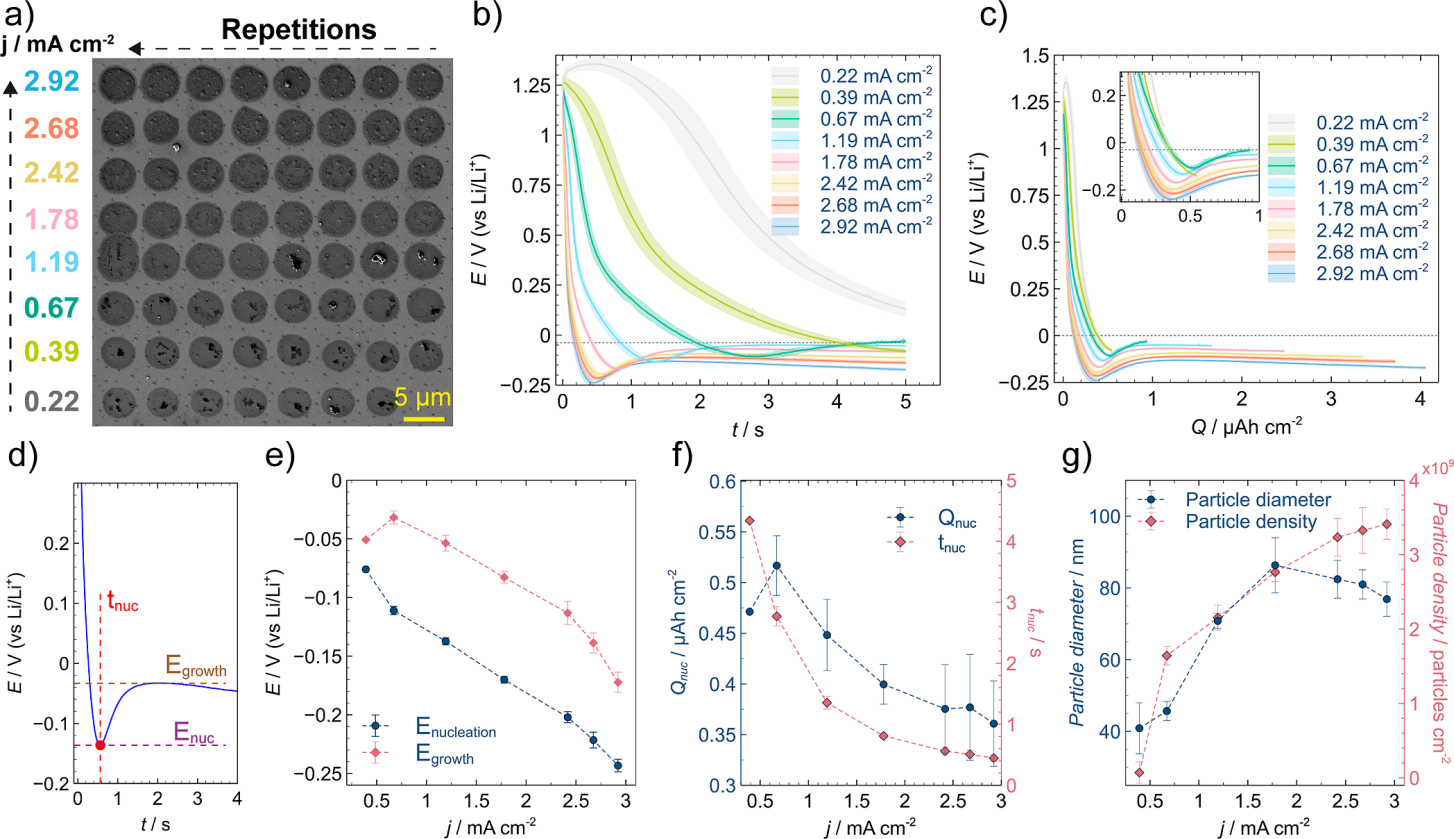
(a) SEM image of the footprints left after the SECCM combinatorial galvanostatic experiment conducted using a pipet with a diameter of ca. 6 μm. Each row represents 8 repetitions for each individual current density (from 0.22 to 2.92 mA cm^–2^); each experiment conducted for 5 s. (b) Galvanostatic (*E-t*) curves recorded for the SECCM combinatorial experiment with (c) representing the same data as (*E-Q*) curves. (d) Schematic representing relevant parameters in the galvanostatic curve such as the nucleation potential (*E*_nuc_), nucleation time (*t*_nuc_) and growth potential (*E*_growth_). (e) Plot of *E*_nuc_ (blue line) and *E*_growth_ (red line) as a function of the current density (*j*). (f) Plot of *Q*_nuc_ (blue line) and *t*_nuc_ (red line) as a function of the current density (*j*). (g) Plot of particle diameter (blue line) and particle density (red line) obtained from SEM as a function of the current density (*j*).

We now demonstrate SECCM/IRM as a powerful high-throughput combinatorial technique to accelerate mechanistic understanding of Li metal plating under a broad set of experimental conditions. A programmatically controlled SECCM pipet probe (ca. 6 μm diameter, Figure S2b) was used to perform combinatorial galvanostatic Li plating at different locations of the thin-film Au electrode. 63 individual measurements were conducted in total as a proof-of-concept by applying 8 different current densities for 5 s with 8 repetitions for each current density (only 7 for the smallest current density), which provided robust statistical analysis. A fraction of this experiment was captured as an IRM movie in Movie S2. The footprints left by this combinatorial Li plating experiment are depicted in Figure 3a, also indicating the average current density applied for each set of repetitions (from 0.22 to 2.92 mA cm^–2^). We calculated the current densities using the geometric size of each SECCM footprint as measured by SEM.

Figure 3b–c show the average galvanostatic responses for each set of conditions, as a function of time (*E-t* curve) or charge (*E-Q* curve), while Figure S10 displays all the individual curves (i.e., repetitions), providing a view of experimental variation. The recorded data reveal typical features of galvanostatic Li plating, as illustrated in Figure 3d. The potential drops initially toward more negative values, during which the SEI and Li_*x*_Au_*y*_ alloys form. Then, a turning point called the nucleation potential (*E*_nuc_) is reached, considered to be the point where the energy applied is enough to overcome the thermodynamic barrier of forming a critical cluster of atoms.^67^ After *E*_nuc_, the potential shifts slightly positive until it reaches a quasi-plateau value called growth potential (*E*_growth_), where Li nuclei are believed to grow. *E*_growth_ is more positive than *E*_nuc_ since Li deposition on already formed Li nuclei is more thermodynamically favorable than creating new nuclei on the heterogeneous metal.^67^

For our experimental time scale (5 s), the potential did not reach *E*_nuc_ for the lowest current density (0.22 mA cm^–2^). SEM images did not reveal any LiNPs in this case (Figure S11), which demonstrates that the charge was only consumed for SEI and Li_*x*_Au_*y*_ formation. The second lowest current density presented a particular case where only one of the repetitions reached *E*_nuc_ (Figure S10b), resulting in the observation of ca. 200 LiNPs (Figure S12). Another two repetitions at this current density (Figure S12) led to the formation of a small number of LiNPs (specifically, 24 and 52), despite the potential not reaching a local minimum (the critical *E*_nuc_ value). This finding reveals that Li nucleation starts well before the potential reaches *E*_nuc_, which has been previously considered as the point where the nucleation thermodynamic barrier is surpassed to form clusters of atoms.^67^ Indeed, a turning point in the potential–time profile requires the Li^+^ flux due to the nucleation and growth processes to become sufficiently large, i.e. that the process becomes relatively facile compared to other competing processes. Our work shows that it is a lagging indicator, as we consider further herein.

All other higher current densities showed qualitatively similar behavior, reaching both *E*_nuc_ and *E*_growth_ (a plateau) for all repetitions, and displaying a clear relationship between the current density and these potentials as shown in Figure 3e, and as previously reported.^67^ The time taken to reach the *E*_nuc_ value (*t*_nuc_) decreased with the current density (Figure 3f, red line), as expected, since the SEI and Li_*x*_Au_*y*_ alloy should take less time to form at higher current densities. However, the charge required to reach *E*_nuc_ (*Q*_nuc_) also decreased with the current density (Figure 3f, blue line). This phenomenon has been previously observed on Cu surfaces in absence of alloy formation,^67^ and is thus assumed to be controlled by the SEI formation.

The different electrochemical response also led to a different coverage and size of LiNPs across the electrode surface as revealed by SEM imaging (Figures S11–S18). The particle density increased with the current density (Figure 3g, red line) as expected from classical nucleation theory,^67−70^ because the resulting increasingly cathodic potential significantly drives the nucleation process. The particle size showed a volcano-type relationship with the current density (Figure 3g, blue line), but the corresponding increase in charge passed (time kept constant at 5 s) complicates this analysis, and a better comparison can made if the charge is kept constant (as addressed below). Nonetheless, an interesting finding was the initial increase in mean diameter of LiNPs from 42 ± 15 to 85 ± 30 nm for current densities from 0.39 to 1.78 mA cm^–2^, followed by a slight decrease in mean diameter to 76 ± 32 nm at 2.92 mA cm^–2^. This is attributed to the presence of many smaller LiNPs in the center of the SECCM probed area, which became increasingly populated at higher current densities, in contrast to being less populated at lower current densities. We suggest these spatial differences in Li deposits are due to the interplay between nucleation kinetics and mass transport rate limitations at different current densities (vide infra).

IRM also provided a general way to follow the coverage of Li deposits across the combinatorial experiment, and a good correlation was obtained between the applied current density and the IRM average intensity (Figure S19).

### Li Plating for Different Current Density but Constant Charge

**Figure 4:**
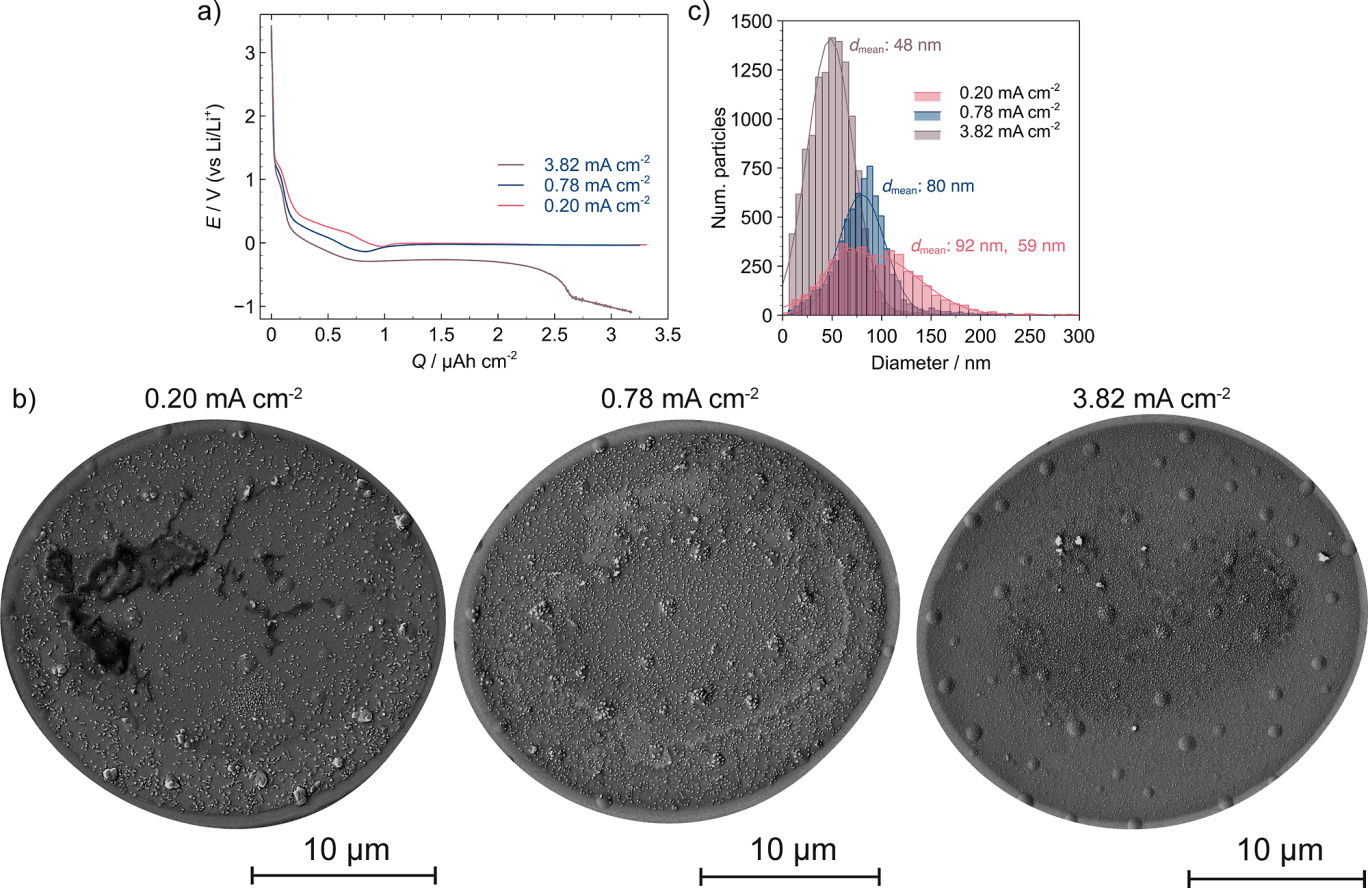
(a) Galvanostatic (*E-Q*) curves recorded for the SECCM galvanostatic experiments conducted using a pipet with a diameter of ca. 10 μm at 0.20 mA cm^–2^ for 60 s, 0.78 mA cm^–2^ for 15 s, and 3.82 mA cm^–2^ for 3 s. (b) SEM images taken at typical footprints after each of the SECCM galvanostatic experiments. Zoomed-in SEM images are shown in Figure S20. (c) Histogram of LiNP diameters of the footprint region from the SEM images. Fitting lines represent a Gaussian distribution.

A further set of SECCM experiments was carried out with a pipet of ca. 10 μm diameter to gain additional spatial information on Li plating dynamics by *in situ* IRM. We evaluated the effect of three current densities (0.20, 0.78, and 3.82 mA cm^–2^) for a variable experimental time (60, 15, 3 s, respectively) to reach a constant final state of charge (*Q* ≈ 3.2–3.3 μAh cm^–2^). Figure 4a shows the galvanostatic responses for this set of experiments along with the corresponding postexperiment SEM images (Figure 4b, zoomed in images in Figure S20). Correlative SIMS imaging is shown in Figure S21. LiNPs covered the electrode surface in all cases, even for the lowest current density (note the time scale was longer than for the previous combinatorial experiment). The galvanostatic response displayed similar trends in *E*_nuc_ and *E*_growth_ (Table 1) to those obtained for the combinatorial experiment, although the potential shifted toward even more negative values for the experiment at 3.82 mA cm^–2^ when the charge exceeded 2.6 μAh cm^–2^. In the first instance, one may attribute this behavior to significant depletion of Li^+^ near the electrode surface. Indicatively, this highest current density is the only case in this set of experiments that surpassed the steady-state Li^+^ mass-transport limiting current under SECCM conditions, estimated to be ca. 1.3 mA cm^–2^ using analytical expressions, assuming diffusion only.^71^ In practice, near 70 s is required to achieve steady-state conditions^71^ (if we define 10% above the limiting current as being sufficiently close to the true steady-state current).^72^ Therefore, Li^+^ depletion on the electrode surface does not occur, which agrees with a consistent rate of Li deposition recorded by IRM for the last 0.5 s of this experiment when the potential already shifted (Figure S22).

**Table 1:** Summary of Electrochemical Parameters and Properties of LiNPs Obtained for the Three SECCM Galvanostatic Experiments

*j* (mA cm^–2^)	*E*_nuc_ (V)	*E*_growth_ (V)	*Q*_nuc_ (μAh cm–2)	*t*_nuc_ (s)	particle density (NPs cm–2)	mean diameter (nm)	Li deposited (fmol)	Faradaic efficiency (%)
0.20	–0.058	–0.004	0.98	17.7	1.1 × 10^9^	92 ± 45; 59 ± 14	91.7	19.7
0.78	–0.135	–0.022	0.85	3.8	1.7 × 10^9^	80 ± 23	70.9	15.2
3.82	–0.292	–0.267	0.82	0.8	2.5 × 10^9^	48 ± 23	49.2	10.6

The analysis of particles by SEM again demonstrates the effect of the current density on particle size and density (Table 1), and also local effects between the edge and center of the SECCM probed area (discussed below). Particle densities were 1.1 × 10^9^, 1.7 × 10^9^, and 2.5 × 10^9^ particles cm^–2^ for 0.20, 0.78, and 3.82 mA cm^–2^, respectively. The mean diameters for the LiNPs were 48 ± 23 and 80 ± 23 nm for the 3.82 and 0.78 mA cm^–2^, respectively, whereas the lowest current density resulted in a double distribution with mean diameters of 92 ± 45 and 59 ± 14 nm as shown in Figure 4c. This demonstrates a superior coverage with smaller LiNPs for higher current densities, which could lead to a more uniform plating at longer time scales.^73,74^ The amount of deposited Li and the faradaic efficiency, assuming ideal spherical nanoparticles, decreased with increasing current densities (Table 1). This observation suggests that conditions where particle growth is promoted over nucleation (low overpotentials) might lead to a higher amount of deposited Li under a similar state-of-charge.

### Spatiotemporal Dynamics of Li Plating Monitored by *In Situ* IRM/SECCM

**Figure 5:**
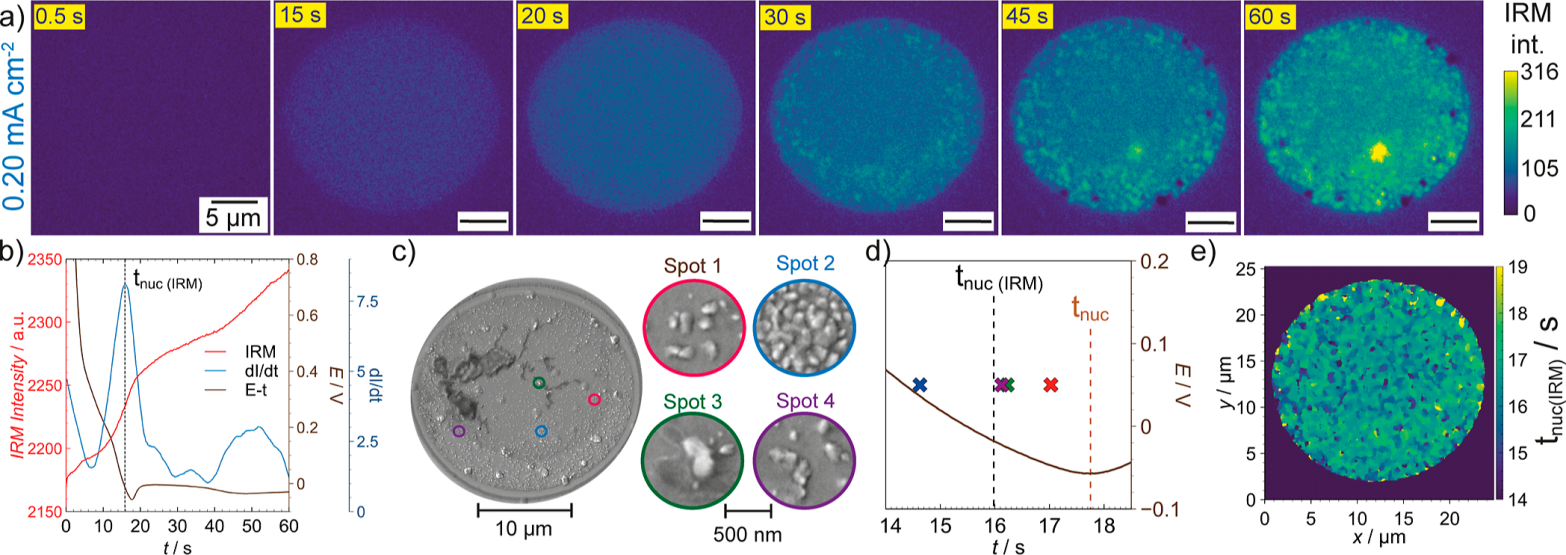
(a) Selected frames for the IRM movie corresponding to the SECCM galvanostatic experiments at 0.20 mA cm^–2^ for 60 s. SECCM pipet diameter was ca. 10 μm. The colorbar on the right represents the IRM intensity. (b) Galvanostatic *E-t* curve (brown line), IRM intensity (red line), and time derivative of the average IRM intensity (d*I*/d*t*) (blue line) over the time of the SECCM galvanostatic experiment in (a). The dashed line indicates the nucleation time from the IRM measurement, *t*_nuc(IRM)_. (c) SEM image of the same experiment showcasing specific areas where the nucleation of a series of LiNP clusters was studied by the IRM analysis. (d) Galvanostatic *E-t* curve (dark brown solid line) and IRM nucleation times obtained for each of the selected clusters shown in (c). The dashed black line indicates the average nucleation time from the IRM measurement, *t*_nuc(IRM)._ And the light brown dashed line indicates the electrochemical nucleation time, *t*_nuc_. (e) Spatially resolved map of Li nucleation times obtained from IRM analysis, *t*_nuc(IRM)_.

The IRM movies (Movies S3–S5) recorded synchronously with the SECCM experiment were used to visualize the spatiotemporal dynamics of Li plating and to reveal the current density-dependent behavior of the growth dynamics. Figure 5a shows a series of frames for the lowest current density (0.20 mA cm^–2^), with a longer sequence in Figure S23. A quite homogeneous IRM intensity across the surface was observed up to 20 s, which is ascribed mainly to the formation of the SEI layer and Li_*x*_Au_*y*_ alloys. The formation of these structures was unaffected by any spatial considerations such as edge vs center effects, which suggests that they are determined by surface-controlled kinetics. After ca. 20 s (note that *t*_nuc_ is 17.7 s in this case), spatial heterogeneities in IRM intensities start to be visualized across the electrode surface related to differences in the nucleation and growth of LiNPs.

There are two major features in the IRM data. First, the IRM intensity increases (shown in yellow color in the images) as a result of the growth of LiNPs. Some hotspots are detected, particularly in the last frame in Figure 5a, where a high density of particles is deposited, as observed from colocated SEM analysis in Figure S24 and spot 2 in Figure 5c. Second, when the thickness of some particles increases significantly, the IRM intensity drops (observed in the image by darker blue spots), which is a known phenomenon in IRM and depends on the nature of the interference phenomena (constructive or destructive).^59,75^ These cases were mainly observed on Au protrusions found at the edges of the probed area, where bigger Li particles were deposited as discussed below.

Tracking the evolution of IRM intensity over time offers a powerful approach for examining Li nucleation at early stages. Figure 5b shows the time derivative of the average IRM intensity (d*I*/d*t*) throughout the experiment, which reveals a local maximum in d*I*/d*t* just prior to the potential reaching *E*_nuc_. The time where this maximum occurs is designated as the IRM nucleation time, *t*_nuc(IRM)_. The same behavior is confirmed for other experimental durations and current densities (Figure S25). This peak-shaped response in d*I*/d*t* is attributed to a variation in the rate of change of IRM intensity, resulting from the onset of Li plating, which leads to a change in the surface refraction index from deposited Li metal. The fact that *t*_nuc(IRM)_ occurs before *t*_nuc_ aligns well with our findings above in the combinatorial experiment (at 0.39 mA cm^–2^) that LiNPs form before the time reaches *t*_nuc_ (proven by SEM images, Figure S12). The IRM analysis thus allows for the detection of Li plating at an earlier stage than found in the electrochemical data, and clearly show that *E*_nuc_ is a lagging indicator of nucleation since the turning point in the potential–time profile appears after nucleation (and to some extent growth) has occurred (vide supra).

This analysis can be used to detect nucleation of different LiNPs across local regions of the electrode surface. We selected four regions of 14 × 14 pixels (ca. 560 × 560 nm^2^) in the IRM images, with the corresponding locations and LiNP clusters in the SEM images shown in Figure 5c. Some of these LiNP clusters displayed *t*_nuc(IRM)_ values close to the average value across the whole area, but *t*_nuc(IRM)_ was ca. 1.5 s earlier for some selected clusters (Figures 5d and S26). This observation indicates that the nucleation of these specific clusters occurs at a lower overpotential, i.e., *E*_nuc_ is different for different nuclei and locations. The spatially resolved map of *t*_nuc(IRM)_ (Figure 5e) clearly show that the nucleation time varies across the probed area, following a normal distribution (Figure S27). However, there is no strong connection between local *t*_nuc(IRM)_ values in this map and the IRM intensity or the size of LiNPs.

**Figure 6:**
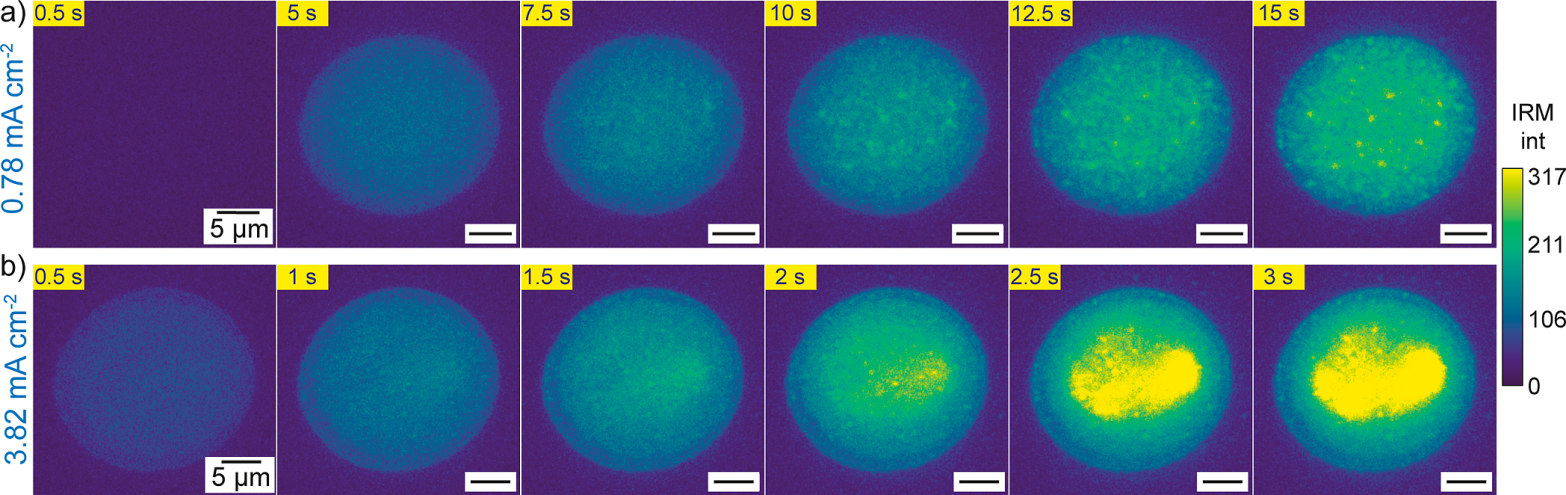
Selected frames for the IRM movies corresponding to the SECCM galvanostatic experiments conducted, using a pipet with a diameter of ca. 10 μm, at (a) 0.78 mA cm^–2^ for 15 s, and (b) 3.82 mA cm^–2^ for 3 s. The colorbar on the right represents the IRM intensity.

The spatiotemporal behavior was different for the intermediate and high current densities. For 0.78 mA cm^–2^, there was less spatial heterogeneity in the IRM movie (Movie S4) as shown in selected frames in Figure 6a (full sequence in Figure S28), which agrees well with the postexperiment particle analysis by SEM as discussed earlier (Figure 4b). A slightly increased IRM signal was observed for the bigger particles that grew on the surface protrusions, as discussed below. For 3.82 mA cm^–2^, it was possible to detect the deposition of the higher density of particles in the center of the SECCM meniscus by IRM (Movie S5 and Figure 6b), as also observed in the postexperiment SEM image (Figure 4b). Interestingly, there are some regions within the center of the probed area with hot spots where the IRM intensity was particularly higher, which is a feature difficult to detect from the SEM image when many small LiNPs are present. IRM thus provides a complementary and dynamic view of spatial heterogeneities that, together with correlative high-resolution SEM analysis, result in a more complete picture of Li plating across the electrode surface.

### Edge Effects

**Figure 7:**
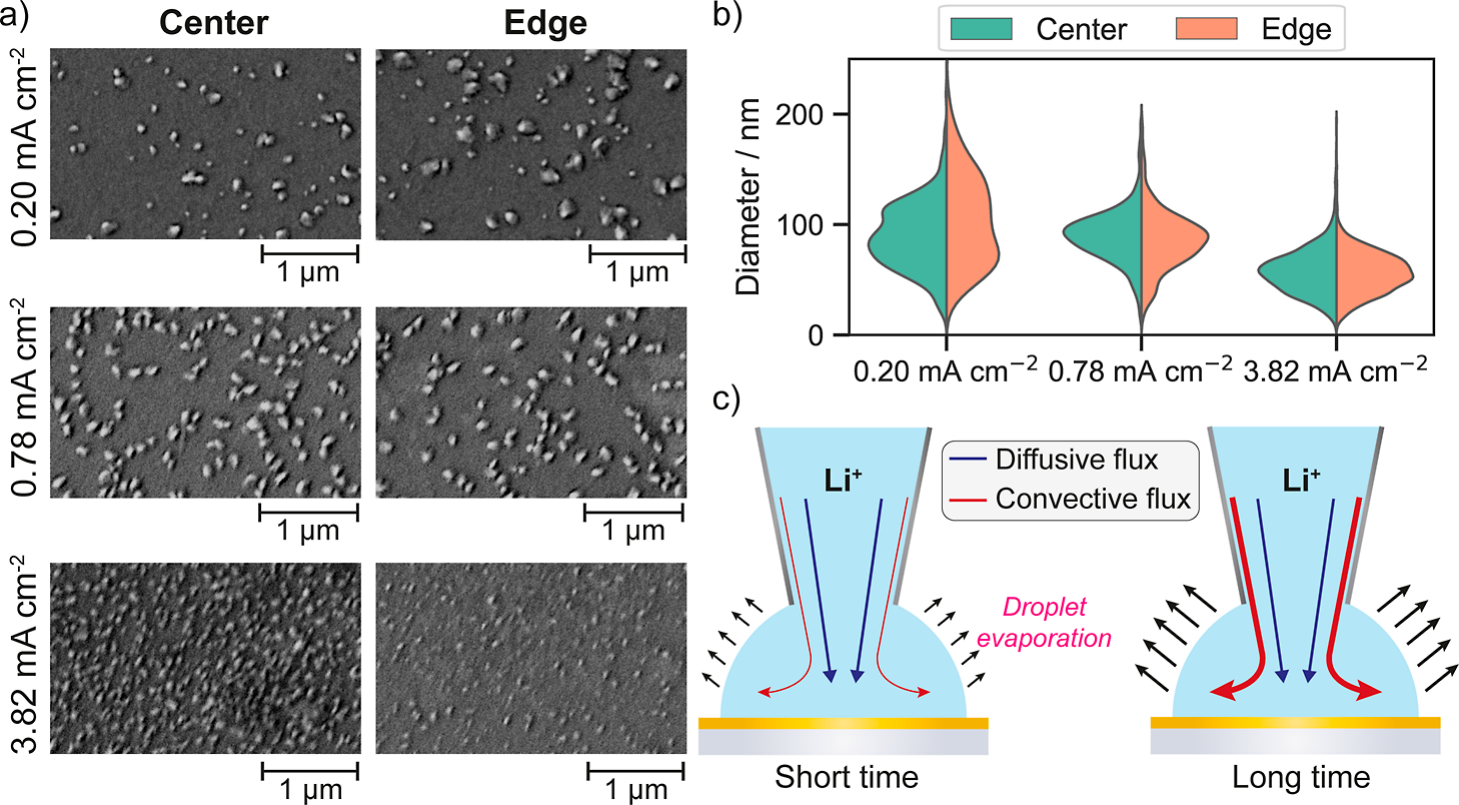
(a) Enlarged SEM images of local regions near the center or edge of the probed area from each of the galvanostatic experiments (0.20, 0.78, and 3.82 mA cm^–2^). (b) Distribution of LiNP diameters observed at the center and edge of the probed area. (c) Schematic representation of diffusive and convective fluxes dominating at short and long experimental times within a SECCM meniscus. Note that line thickness represents qualitatively the flux rate.

The SECCM configuration allows us to compare information from the edge and the center of the probed area. Figure 7a shows SEM images of local regions for the three different current densities examined (0.20, 0.78, and 3.82 mA cm^–2^, for the same final state of charge) whereas Figure 7b presents the distribution of LiNP diameters by region. Larger LiNPs were found at the edges for the lowest current density (0.20 mA cm^–2^), indicating a faster growth rate for LiNPs that nucleate at the edges (2.2 vs 1.4 nm s^–1^). Tentatively, we suggest this might be due to solvent evaporation of the SECCM meniscus (Figure 7c) that induces a convective flux toward the meniscus edge to compensate for the evaporated liquid,^76−78^ with the evaporation flux increasing with experimental time.^78^ Despite the high boiling point and low vapor pressure of PC (3.066 Pa at 25 °C),^79^ some evaporation of the microscale meniscus is reasonable at these experimental time scales (60 s). This is evident from the rapid evaporation of the droplet left after the SECCM probe retracted (Figure S29), although the rate may be slower when the probe is in contact. The meniscus edge is also a liquid wedge topological defect which, in general, may promote nucleation and growth,^80^ and such effects will be most manifested at low driving force. In general, the density of LiNPs was only slightly higher at the edges (1.22 × 10^9^ vs 1.08 × 10^9^ particles cm^–2^), indicating that the edge-center context has a relatively minor impact on nucleation at low current density.

A more uniform size distribution and particle density across the probed area were observed at the intermediate current density (0.78 mA cm^–2^). This suggests that intermediate current densities may represent an optimal range where both nucleation and growth are not affected by spatial effects, at least up to this experimental time. For the highest current density (3.82 mA cm^–2^), the size of the LiNPs at the center and edges was relatively homogeneous (Figure 7b), but a higher density of particles at the center of the probed area was detected (3.2 × 10^9^ vs 1.5 × 10^9^ particles cm^–2^). Under these high-current conditions, nucleation readily takes place upon Li^+^ reaching the surface, directly under the pipet tip, which temporarily shields the edge of the meniscus from significant Li^+^ delivery. However, increased growth toward the edge of the meniscus is also observed at longer time scales under high current densities (Figure S30). This finding further corroborates the previous observation under low current densities that suggests that a different mass transport regime directs the flux of Li^+^ toward the meniscus edge at extended experimental times, likely convective fluxes generated by meniscus evaporation coupled with more complex mass transport due to the change in the surface topography due to more extensive metal nucleation and growth.

### Role of Local Electrode Topography

**Figure 8:**
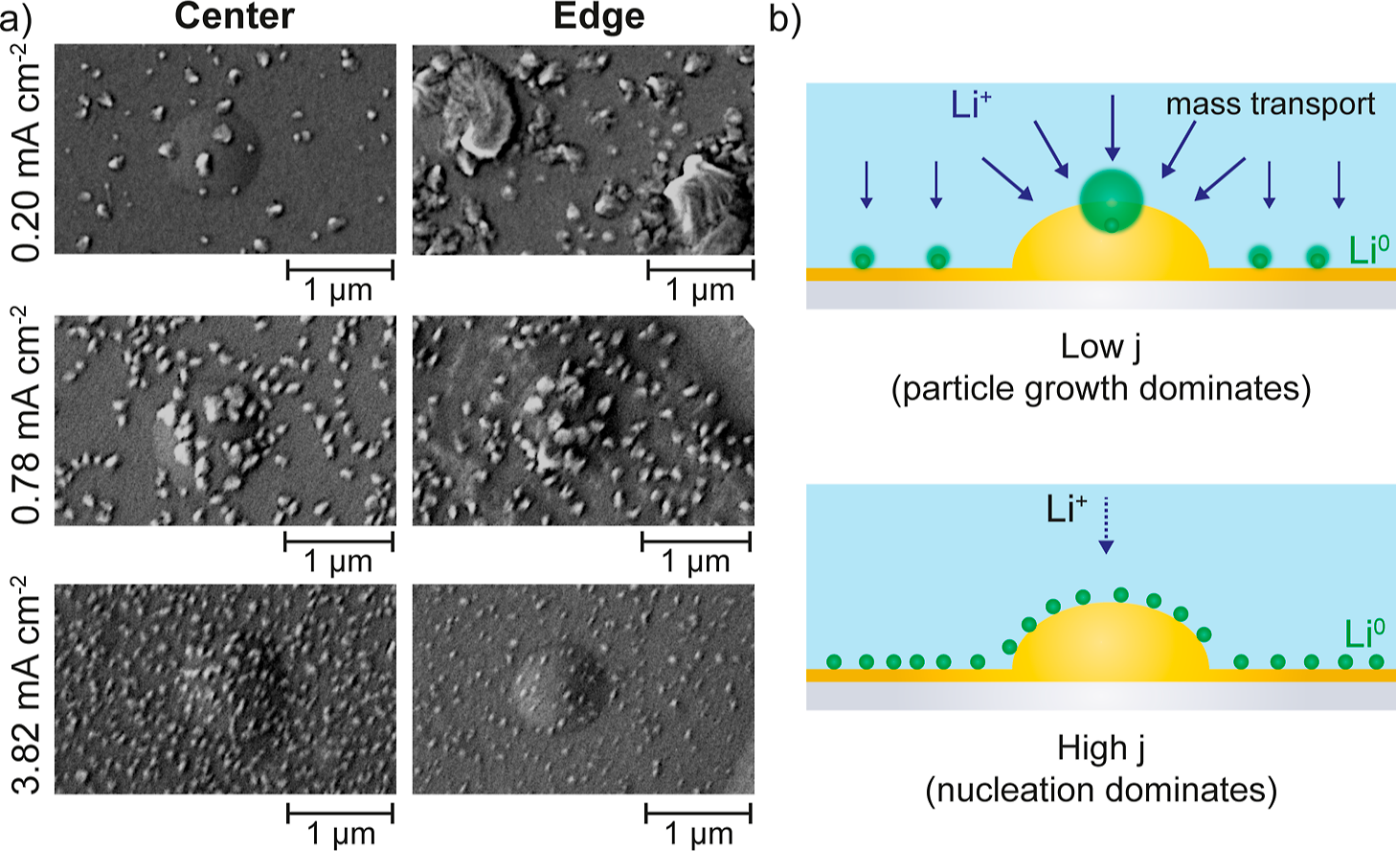
(a) Enlarged SEM images of local regions with protrusions near the center or edge of the probed area from each of the galvanostatic experiments (0.20, 0.78, and 3.82 mA cm^–2^, for the same final state of charge). (b) Schematic representation of enhanced local fluxes due to hemispherical diffusion toward a surface protrusion, prevalent at low current density (top), and when the process is dictated by nucleation over mass transport at high current density (bottom).

Turning our focus toward the electrode surface topography, we last analyze the impact of topographical features on Li growth, which is presently an important matter of discussion.^20,70^ It has been suggested that curvature around topographical features leads to a higher electric field, attracting more Li^+^ ions,^9^ and it has also been proposed that hemispherical Li^+^ ion diffusion around such features accelerates Li growth.^81^ Herein, we present experimental evidence illustrating the effects of locally heterogeneous lithiophilic surfaces as the thin-film Au electrodes presented nanoscale protrusions (Figure S1). Figure 8a shows SEM images of Li NPs on these protrusions at the center and edges of typical SECCM footprints. These images demonstrate the clear influence of both current density and protrusion location on the growth of Li structures. For the lowest current density, the LiNPs in protrusions grew faster than those on a nearby flat surface, but only when the protrusions were located at the meniscus edge. The contribution of a convective flux toward the edge, as discussed earlier, provides faster delivery of Li^+^ ions and growth of the LiNPs. The effect of protrusions was only minor at the meniscus center. For the intermediate current density, the local effect of protrusions is still evident, but the differences in the size of LiNPs compared to those on flat regions are less pronounced. In addition, the edge versus center effect is no longer as noticeable. For the highest current density, the protrusions do not accelerate the growth rate of LiNPs, resulting in similar sizes observed across the entire surface. Under these conditions, Li plating remains primarily controlled by nucleation, and local differences in phenomena related to mass transport, such as particle growth, are not evident.

These findings indicate that Li growth on uneven current collectors depends not only on the local mass transport regimes created by those heterogeneous features—which is expected to be increased by contributions from radial diffusion where the reaction on the protrusion is favored compared to the surrounding surface (at lower driving force and at the meniscus edge) (Figure 8b, top)—but also on the global mass transport rates across the entire electrode. In addition, the particular stage within the nucleation/growth process plays a significant role in determining the relative impact of surface protrusions, inexistent e.g. under high current densities when nucleation still governs (Figure 8b, bottom). This suggests that working under high overpotentials that promote nucleation could be an effective strategy to mitigate the effects of local heterogeneous surface structures.

## Conclusions

Through an *in situ* SECCM/IRM and *ex situ* SEM multimicroscopy approach, we have investigated Li metal deposition and stripping on lithiophilic thin-film Au surfaces. Our findings revealed that Li nucleation and growth are significantly impacted by the interplay between applied current density, local mass transport effects, and local electrode structure, such as protrusions. For the range of current densities studied, intermediate current densities led to a more homogeneous distribution of LiNPs, while higher and lower current densities were subject to mass transport effects resulting in spatial heterogeneities in either the particle size or areal density distributions.

Importantly, our approach enables the *in situ* visualization of inactive Li, and the generation of spatially resolved *CE* maps with nanoscale resolution, which reveals local capacity losses across the electrode surface *cycle-by-cycle*. Additionally, we obtained spatially resolved analysis of the nucleation time by optics, *t*_nuc(IRM)_, providing insights into varying nucleation kinetics, at earlier stages than what is possible by the (lagging) electrochemical data, and identifying specific areas across the electrode surface that promote (or inhibit) Li nucleation.

This work also showcases the potential of correlative electrochemical multimicroscopy in accelerating mechanistic understanding of systems by leveraging the high-throughput capabilities of SECCM for combinatorial screening of electrochemical conditions, also offering robust statistical analysis through repetitions. We present a proof-of-concept involving multiple measurements with different current densities, but the approach can be expanded to various electrochemical techniques and parameters, potentially allowing for the study of metal plating and stripping under thousands of conditions in a single experiment and a short time. The major bottleneck might lie in the *ex situ* acquisition of colocated high-resolution images by electron microscopy, although automated protocols might soon become more widely available.^82^ Nonetheless, our method has strong potential to be complemented by machine learning methodologies,^83^ providing a data-driven prediction of properties from Li deposits by supplying high-throughput experimental data.

Given the significance of metal plating and stripping processes in the battery field and beyond, we believe that these high-throughput multimicroscopy approaches based on SECCM will yield substantial insights into the complex mechanism of nucleation and growth across a variety of electrochemical interfaces and materials. A future objective is to bridge the gap between the insights obtained at the nano- and microscales from these techniques and the practical performance of full-cell batteries.

## Methods

### Reagents and Materials

A 50 mM lithium hexafluorophosphate (LiPF_6_) solution in PC was used for all experiments. This concentration was chosen to prevent excessive Li deposition and to enable the detection of nucleation and growth of small Li nanoparticles during electrodeposition. Additionally, it avoids significant electrolyte residue covering the surface for *ex situ* characterization, issues that might arise with higher electrolyte concentrations. The working electrolyte was prepared from a commercial battery-grade 1.0 M LiPF_6_ in PC solution (Sigma-Aldrich, HF < 50 ppm, H_2_O < 15 ppm), which was diluted in PC (>99%, acid <10 ppm, H_2_O < 10 ppm).

Silver (Ag) wires with a diameter of 0.25 mm and a purity of 99.99% (Goodfellow) served as quasi-reference counter electrodes (QRCEs). The potential of the Ag QRCE was converted to the Li/Li^+^ scale following calibration against the IUPAC-recommended Fc/Fc^+^ redox process,^84^ as described previously.^50^

To prepare thin-film gold (Au) electrodes, metal deposition was carried out on glass coverslips (⌀22 mm, thickness: 0.16–0.19 mm; Academy) using an SVS 8 pocket electron beam evaporator (Scientific Vacuum Systems). An initial 2 nm thick titanium (Ti) film was deposited at a rate of 0.5 Å s^–1^, followed by a 20 nm thick Au film at 1.5 Å s^–1^. The deposition process was conducted under a pressure of 7 × 10^–7^ mbar and at a room temperature of 24 °C, without cooling the sample stage.

### Characterization of the Thin-Film Au Electrodes

X-ray photoelectron spectroscopy (XPS) was employed to determine the surface composition of the thin-film Au electrodes. The XPS data were collected at the Photoemission Research Technology Platform, University of Warwick. The sampled investigated in this study was attached to electrically conductive carbon tape, mounted on to a sample bar and loaded into a Kratos Axis Ultra DLD spectrometer which possesses a base pressure below 1 × 10^–10^ mbar. XPS measurements were performed in the main analysis chamber, with the sample being illuminated using a monochromated Al Kα X-ray source (*h*ν = 1486.7 eV). The measurements were conducted at room temperature and at a takeoff angle of 90° with respect to the surface parallel. The core level spectra were recorded using a pass energy of 20 eV (resolution approx. 0.4 eV), from an analysis area of 300 × 700 μm. The work function and binding energy scale of the spectrometer were calibrated using the Fermi edge and 3d_5/2_ peak recorded from a polycrystalline Ag sample prior to the commencement of the experiments. The Au 4*f* core level data were analyzed in the CasaXPS package using a Shirley background and a DS(0.03, 320) line shape. Figure S1a–b show the survey and the high-resolution Au 4*f* spectra, respectively. The Au film was found to consist exclusively of Au(0), whereas the presence of some carbon and oxygen groups is likely due to ambient exposure of the samples.

Atomic force microscopy (AFM) was carried out to extract the topography of the thin-film Au electrodes using a Dimension Icon microscope (Bruker) in PeakForce tapping mode with tips of silicon on nitride lever (SCANASYST-AIR, Bruker). Scans were captured with 256 points per line at 0.5 Hz across 5 × 5 μm^2^ areas. AFM images were analyzed with the Gwyddion software (v2.62, Czech Metrology Institute). Figure S1c shows the surface topography of the thin-film Au electrodes, revealing local regions with the presence of hemispherical protrusions of nanoscale dimensions. Specifically, these protrusions typically measured between 400 and 1000 nm in diameter, with heights ranging from 10 to 40 nm (Figure S1d). Regions without these protrusions were flat and uniform, with a surface roughness significantly lower than 1 nm.

### SECCM with IRM

#### Pipette Fabrication

Single-channel pipettes were fabricated using borosilicate filamented capillaries (GC120F-10, Harvard Apparatus) with an outer diameter of 1.2 mm, an inner diameter of 0.69 mm and a length of 100 mm. A CO_2_-laser puller (P-2000, Sutter Instruments) was employed for this purpose. Pipettes with tip openings of ca. 10 μm (verified by optical microscopy, Figure S2a) and ca. 6 μm (Figure S2b) were produced using the specified pulling parameters: HEAT 350, FIL 3, VEL 22, DEL 220, PUL - (10 μm), and HEAT 350, FIL 3, VEL 24, DEL 220, PUL - (ca. 6 μm). We used relatively large pipet diameters to ensure a considerable region of the surface could be visualized by *in situ* optical imaging.

#### SECCM/IRM Setup

A custom-built SECCM workstation assembled on top of an inverted microscope (openFrame, Cairn Research) enabled simultaneous SECCM and IRM measurements.^56^ This workstation was housed within an argon-filled glovebox (MBraun MB200B/MB20G) that maintained H_2_O levels below 0.2 ppm and O_2_ levels under 0.1 ppm, as previously reported.^50^ To minimize the impact of mechanical vibrations, the entire setup was placed on a vibration isolation platform (BM-10 minus K Technology).

#### SECCM Measurements

A pipet filled with 50 mM LiPF_6_ in PC was equipped with a Ag QRCE and attached to a 3-axis *xyz* piezoelectric positioner (P-611.3S, NanoCube, Physik Instrumente). The pipet was initially positioned near the electrode surface using coarse manual movement and a *z*-axis picomotor (8303 Picomotor Piezo Linear Actuator, Newport), aided by both an optical camera (PL-B782U, 2X lens, Pixelink) and the inverted microscope in transmission mode (top illumination) using a small torch (Ansmann, 150 lm) placed above the system. To reduce electrical noise, the SECCM setup was covered with a copper woven mesh (60 mesh per inch, 0.166 mm wire diameter, Cadisch Precision Meshes).

Different devices were employed for recording electrochemical data, depending on whether the experiments involved potentiodynamic or galvanostatic measurements. For potentiodynamic measurements (i.e., CV), a custom-built electrometer recorded the surface (WE) current.^85,86^ Contact between the liquid meniscus formed at the pipet tip and the Au surface was detected when the current exceeded a threshold value of 5 pA, while a potential of +1.47 V (vs Li/Li^+^) was applied during pipet approach. For galvanostatic measurements, a custom-built galvanostat was set to the “overload” value (+10 V),^87^ and a detectable decrease of more than 5 V from this value indicated the contact of the liquid meniscus with the substrate electrode surface. Upon detection, pipet movement was immediately halted, and the galvanostatic measurement was recorded. In combinatorial experiments, a hopping-mode protocol was applied as previously reported.^51^ Lateral separation between individual measurements was 11 μm in the *xy* plane, and the retract distance in the *z* plane was 8 μm. Approach, retract, and lateral movement rates were set at 2 μm s^–1^.

Data acquisition and instrument control in SECCM were conducted using a field programmable gate array card (PCIe-7852R, National Instruments) managed by a LabVIEW 2020 (National Instruments) interface operating the Warwick Electrochemical Scanning Probe Microscopy (WEC-SPM) software.^88^ Data was sampled every 10 μs and averaged 256 times, with one extra iteration for transferring data to the computer, resulting in a data acquisition rate of 2.57 ms. Data processing and analysis were performed using a custom-written Python code based on SciPy libraries.^89^ The electrochemical response is presented as current density, determined by the meniscus footprint (wetted area) on the Au electrode, as imaged by SEM.

#### IRM Measurements

IRM images were captured using a CellCam Centro 200 MR camera, recording at a rate of approximately 4.3 frames per second (fps) or 230 ms per frame with 12 bit resolution. Note that the actual rate varies frame by frame, and the real values are used for all the data analysis. Back-illumination was supplied by a multi-LED light source (CoolLED pE-300 Ultra), coupled with a reflective neutral density optical filter (optical density: 2.0; 1% transmission; Edmund Optics). The green channel LED, featuring an intensity peak at a central wavelength of ca. 560 nm according to the manufacturer, was utilized during experiments at an intensity of 10%. The camera was situated inside the glovebox, while the light source was placed externally and connected to a UV-transmitting liquid light guide (2 m long, 3 mm core diameter; CoolLED), which passed through a glovebox feedthrough. A Nikon CFI Plan Apochromat Lambda objective, offering 60× magnification and a numerical aperture of 0.95, was employed. With this setup, we achieved a pixel size for IRM images of about 40 nm. As discussed previously,^59,61^ although resolving the optical features may be limited by diffraction, the detection limit in the IRM configuration is typically lower than the diffraction limit and can reach the order of 10 nm. MicroManager (v2.0) was used to control the microscope system.^90^ A positioning stage, which was mounted on the microscope frame, featured an aperture above the inverted objective to hold the sample securely. Further information on IRM, also referred to as backside absorbing layer microscopy, can be found elsewhere.^61,91^

### SEM and SIMS

SEM and SIMS measurements were performed by a dual focused ion beam and scanning electron microscopy system (FIB-SEM, FEI Scios). Quadruple SIMS (EQS HIDEN Analytical) characterization was performed under high vacuum at a pressure below 5 × 10^–6^ mbar. A 30 keV Ga^+^ ion beam with primary ion beam current of 10 pA (2 sputtering cycles, 1 ms dwell time) was used for imaging the Li distribution. The raster size was 1000 by 1000 pixels, which leads to a practical lateral resolution of about 27 nm. Samples prepared after SECCM were always transferred from the glovebox to the microscope chamber through an airless-transfer kit to prevent side reactions of reactive lithium and SEI components.

### Image Processing Methods

ImageJ (version 2.9.0), an image processing package, was employed to analyze IRM image stacks (i.e., movies) and detect and count lithium nanoparticles in SEM images. Synchronization between IRM and SECCM was achieved by designating the first IRM frame with a detected droplet contact (indicated by a change in optical intensity) as the initial time for SECCM.

For IRM movies, each sequence was cropped to cover an area near the SECCM footprint, and only frames corresponding to the SECCM experiment were considered for analysis. The initial frame (where SECCM landing is detected) served as the background frame and was subtracted pixel by pixel from subsequent frames to highlight changes resulting from the SECCM experiment. A projection method (“Grouped Z project. . .” in ImageJ) was then applied using the average intensity with group sizes of 2 frames. This process produced an image stack with half of the original frames (ca. 460 ms per frame) but with significantly enhanced signal-to-noise rate. Additional noise was removed by applying a Gaussian filter (sigma radius: 1 μm) to each frame.

To detect nucleation times (*t*_nuc_), the raw IRM data without any processing was used. A specific area of interest within the SECCM footprint was selected, and the average IRM intensity (*I*_IRM_) at that selected location was determined. The time derivative (dI_IRM_/dt) was then calculated, a one-dimensional Savitzky–Golay filter was applied using the SciPy library,^89^ and the resulting data was plotted as a function of experimental time. The local maximum as shown in Figure 5b was identified as *t*_nuc(IRM)_. A similar process was repeated pixel-by-pixel across the IRM images in order to represent spatially resolved *t*_nuc(IRM)_ maps.

Coulombic efficiency (*CE*) analysis using IRM data, named *CE*_(IRM)_, involved determining the change in IRM intensity after stripping (*I*_a_) and plating (*I*_c_) on a pixel-by-pixel basis, similar to the representation in Figure S5. The linear relationship between *I*_a_/*I*_c_ and *CE*, as determined from the average IRM response (entire SECCM footprint) and the electrochemical data (Figure S5), was used to calculate pixel-by-pixel *CE*_(IRM)_ values. These values were subsequently represented as spatially resolved *CE*_(IRM)_ maps. Only the area from the SECCM footprint was analyzed, which was extracted through thresholding and masking using the OpenCV python library.

ImageJ was also used for counting and sizing lithium nanoparticles from SEM images, following this protocol: (1) applying a Gaussian filter to smooth the image and enhance particle detection, (2) thresholding for image segmentation into features of interest and background, (3) employing the watershed method to separate overlapping or touching particles, (4) selecting the area of the SECCM footprint, and (5) analyzing particles to count and size the detected nanoparticles in the selected area. Particles larger than 0.1 μm^2^ (ca. 350 nm in diameter) were excluded to minimize false positive, as lithium nanoparticles observed on the surface were smaller. For size calculations, particles were assumed to be quasi-spherical, though some deviation from a perfect spherical shape may be observed in some particles.

## Data Availability

Raw data are available in a Zenodo repository (doi: 10.5281/zenodo.10977531).

## Supporting Information Available

The Supporting Information contains *in situ* IRM movies for various electrochemical experiments (Movies S1–S5), additional data such as the characterization of the thin-film Au electrodes and SECCM pipettes, colocated SIMS maps, colocated SEM images for the combinatorial experiment, and other figures that support the main findings presented in this work (PDF)
Movie S1. Movie showing the IRM intensity variation during the SECCM experiment for 10 voltammetric cycles between +1.53 and –0.21 V vs Li/Li^+^ at a scan rate of 100 mV s^–1^ (AVI)
Movie S2. A fraction of the SECCM combinatorial galvanostatic experiment recorded by IRM imaging (AVI)
Movie S3. Movie showing the IRM intensity variation during the SECCM galvanostatic experiment at 0.20 mA cm^–2^ for 60 s (AVI)
Movie S4. Movie showing the IRM intensity variation during the SECCM galvanostatic experiment at 0.78 mA cm^–2^ for 15 s (AVI)
Movie S5. Movie showing the IRM intensity variation during the SECCM galvanostatic experiment at 3.82 mA cm^–2^ for 3 s (AVI)

## Supplementary Material

nn4c05001_si_001.pdf

nn4c05001_si_002.avi

nn4c05001_si_003.avi

nn4c05001_si_004.avi

nn4c05001_si_005.avi

nn4c05001_si_006.avi
