# Supplementary material for: High-Throughput Combinatorial Analysis of the Spatiotemporal Dynamics of Nanoscale Lithium Metal Plating
Source: ACS Nano. 2024 Aug 13;18(34):23032–46. doi: 10.1021/acsnano.4c05001 (PMC11363218; doi:10.1021/acsnano.4c05001)
Supplement: Supplementary file 1 — nn4c05001_si_001.pdf [file nn4c05001_si_001.pdf]

# SUPPORTING INFORMATION

## High-throughput combinatorial analysis of the spatiotemporal dynamics of nanoscale lithium metal plating

Daniel Martín-Yerga,[1,2*] Xiangdong Xu,[1] Dimitrios Valavanis,[1] Geoff West,[3] Marc Walker,[4] Patrick R. Unwin[1*]

[1]*Department of Chemistry, University of Warwick, CV4 7AL Coventry, UK*

[2]*Department of Chemistry, Nanoscience Center, University of Jyväskylä, 40100 Jyväskylä, Finland*

[3]*Warwick Manufacturing Group, University of Warwick, CV4 7AL Coventry, UK.*

[4]*Department of Physics, University of Warwick, CV4 7AL Coventry, UK*

*Corresponding authors' email:

daniel.martin-yerga@warwick.ac.uk (D.M-Y)

p.r.unwin@warwick.ac.uk (P.R.U)

## ADDITIONAL FIGURES

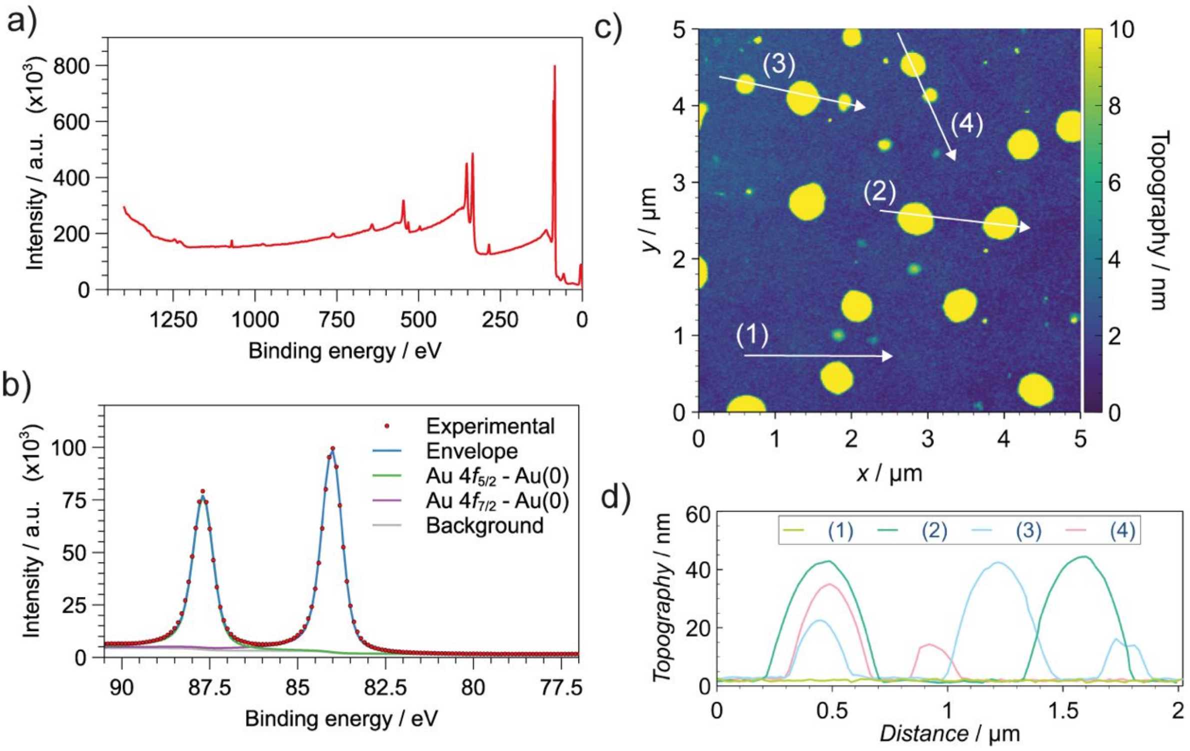


**Figure S1.** (a) XPS survey and (b) high-resolution Au 4*f* spectra obtained for the thin-film Au electrodes. (c) AFM topography of a region of the thin-film Au electrode, and (d) line profiles for specific positions indicated in (c).

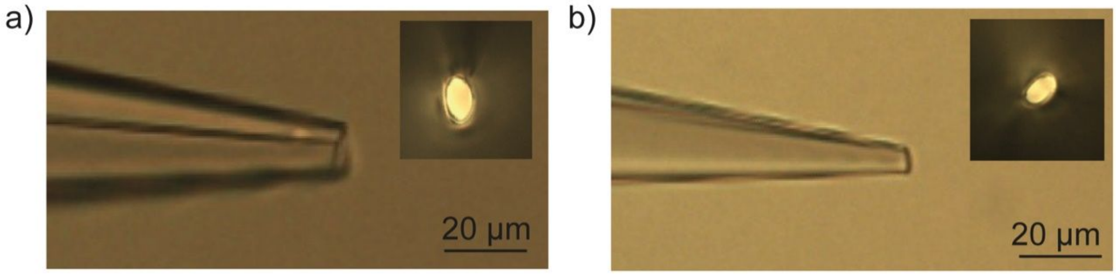


**Figure S2**. Optical microscopy images of micropipettes used in this work: (a) ca. 10 µm diameter, (b) ca. 6 µm diameter.

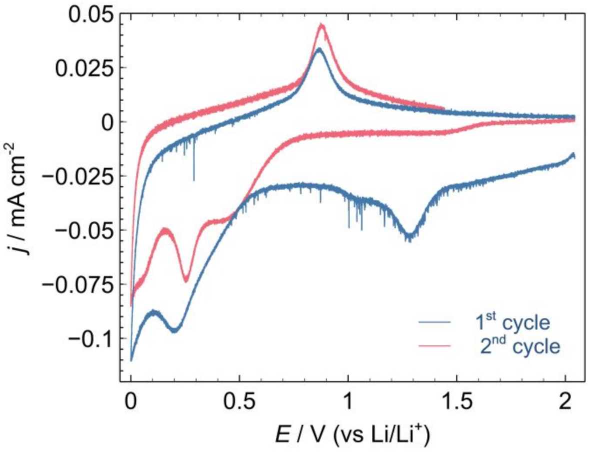


**Figure S3**. Cyclic voltammetry (2 cycles) recorded with a cut-off potential of 0 V vs Li/Li$^+$, where lithium plating should be minimal or non-existent. These measurements were obtained with pipettes of diameter ca. 10 μm filled with 50 mM $LiPF_6$ in PC solution. Scan rate was 25 mV $s^{-1}$.

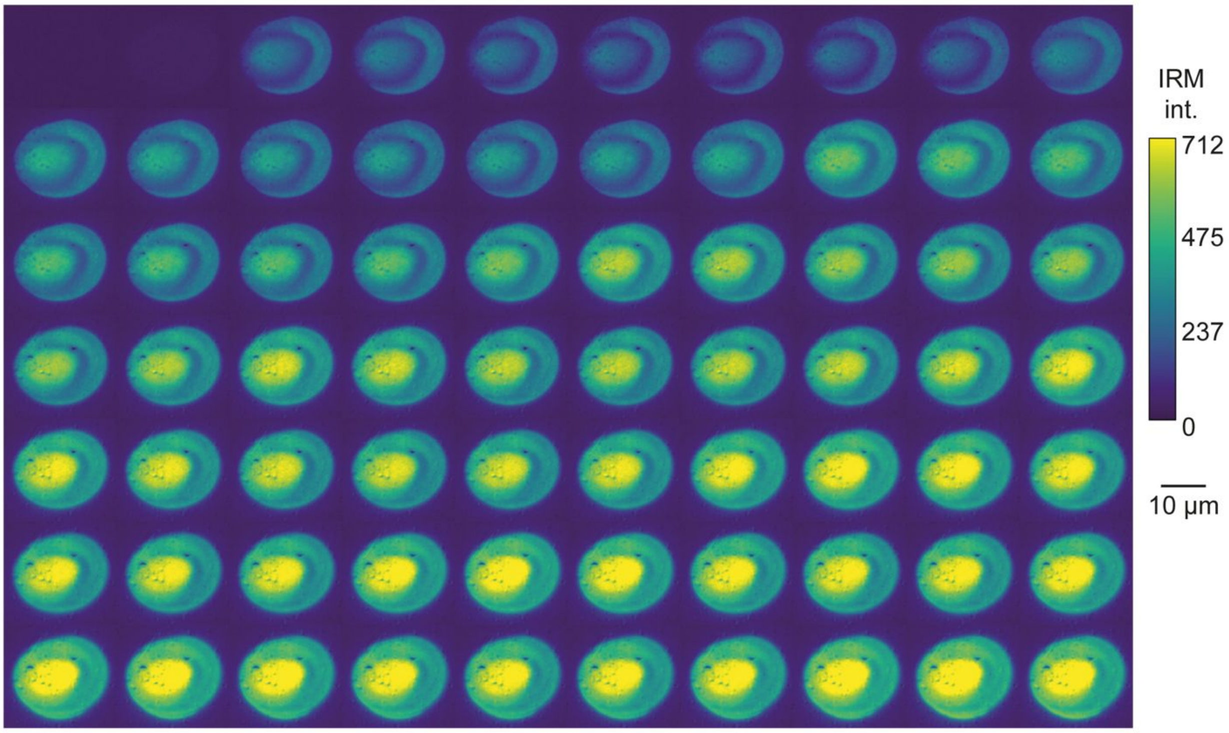


**Figure S4**. A series of frames of the IRM movie corresponding to the SECCM experiment recording 10 voltammetric cycles with a cut-off potential of -0.27 V and a scan rate of 100 mV $s^{-1}$. One frame is shown herein for each ten frames of the full movie (ca. 4.6 s). Scale bar is 10 μm. The voltammetric profile is represented in Figure 2a (main manuscript). The experimental time increases from left-to-right and from top-to-bottom.

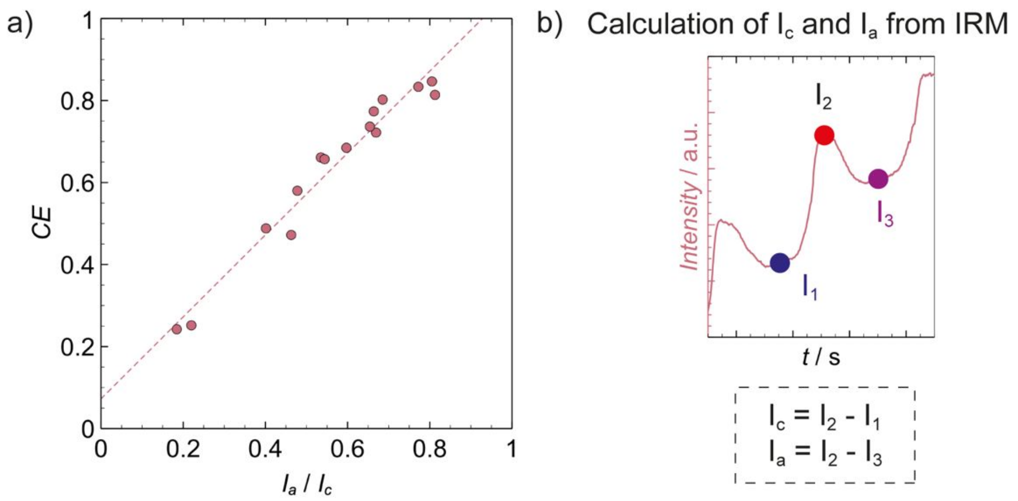


**Figure S5.** (a) Relationship between the coulombic efficiency (*CE*) and the ratio of the change in IRM intensity after stripping ($I_a$) and after plating ($I_c$). A linear relationship was found, with the regression equation given by $CE = 0.0726 + 0.9988(Ia/Ic)$ and an $R^2$ value of 0.96. Data were acquired from two separate SECCM voltametric experiments (10 cycles and 5 cycles) to improve the accuracy of the linear regression. (b) Schematic demonstrating the calculation of $I_a$ and $I_c$ values from the IRM data for a specific voltammetric cycle.

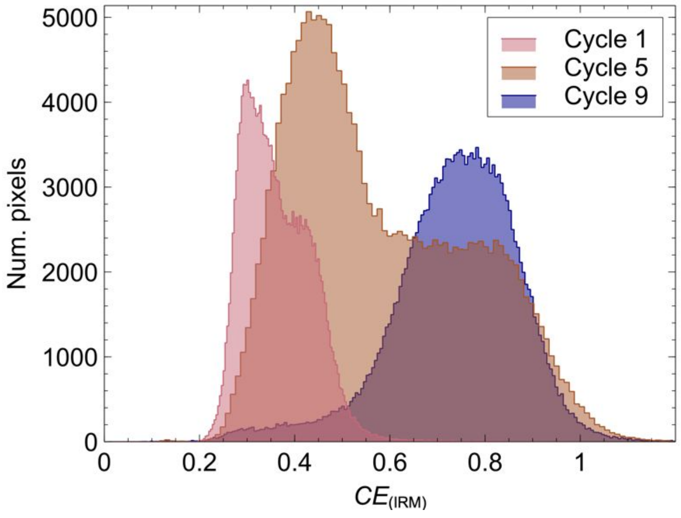


**Figure S6**. Histograms of spatially-resolved coulombic efficiency values extracted from IRM, $CE_{(IRM)}$, for the first, fifth and ninth voltammetric cycles of the SECCM voltammetric experiment.

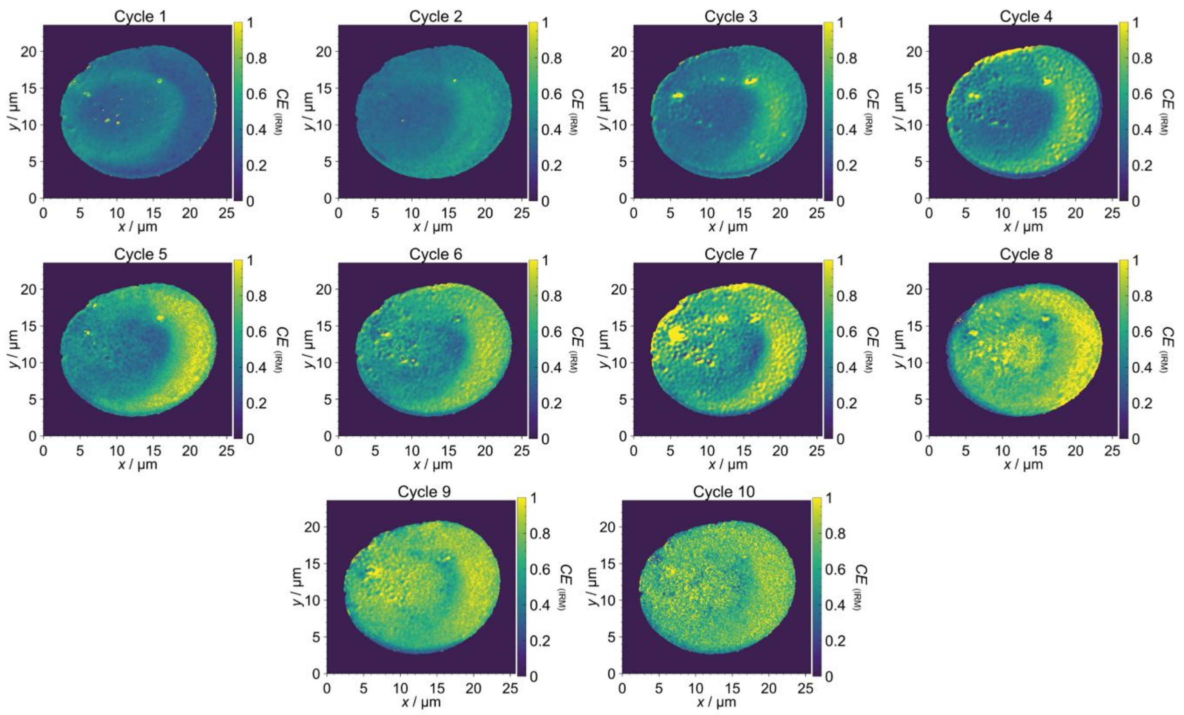


**Figure S7**. Full sequence of cycles of spatially-resolved maps of coulombic efficiency extracted from IRM, $CE_{(IRM)}$, for the SECCM voltammetric experiment.

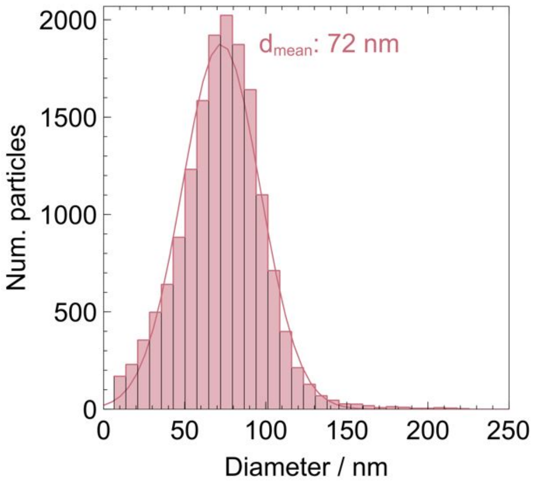


**Figure S8**. Histogram of diameter (d) values for the lithium nanoparticles observed by SEM imaging after the SECCM voltammetric experiment. The fitting line follows a gaussian distribution with mean of 72 nm.

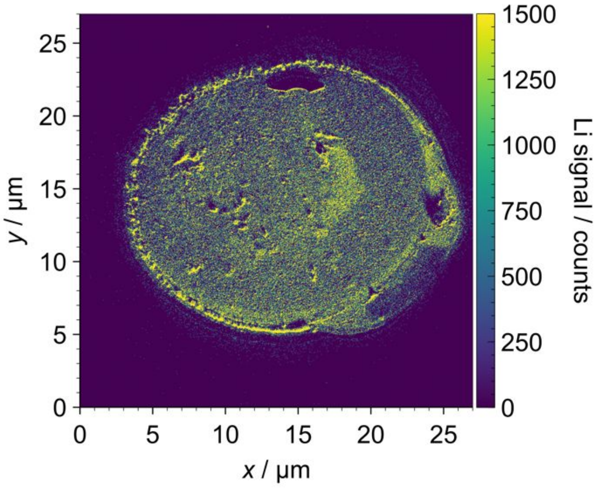


**Figure S9**. Secondary ion mass spectrometry (SIMS) $Li^+$ map of the footprint left after the SECCM voltammetric experiment with 10 cycles of plating and stripping. SIMS analysis confirmed the presence of Li across the SECCM footprint. Limitations of SIMS herein include detecting Li originating from various sources such as deposited Li metal, Li from $Li_xAu_y$ alloys, and rests of electrolyte residue. The residue was not rinsed to preserve the original structure and prevent the removal of Li nanoparticles.

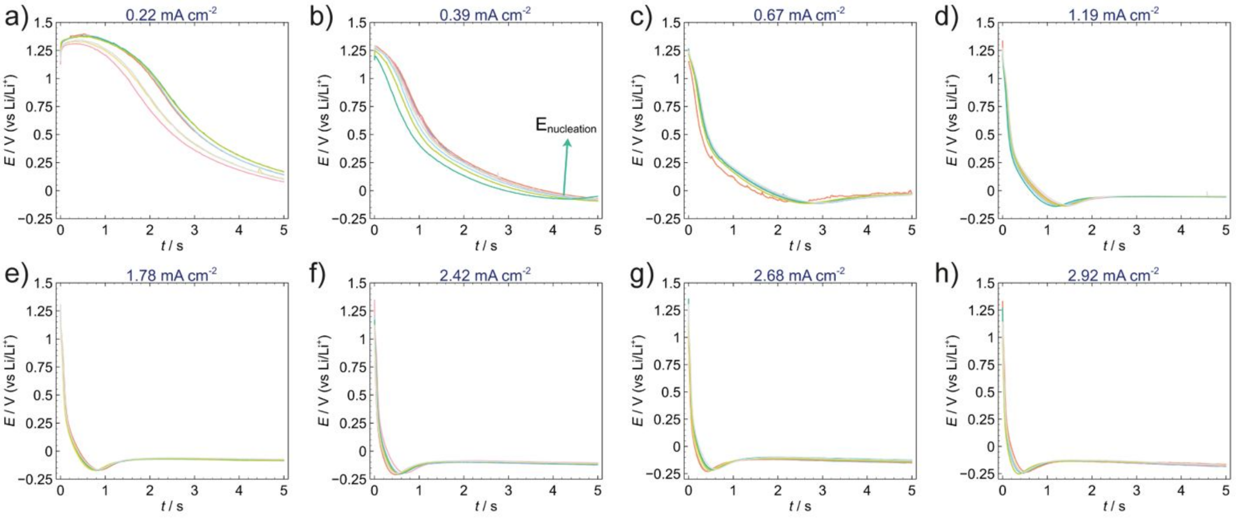


**Figure S10**. All individual galvanostatic (*E-t*) curves recorded for the SECCM combinatorial experiment for each current density. 63 independent measurements were recorded in total (7 repetitions for 0.22 mA $cm^{-2}$ and 8 repetitions for each other current density).

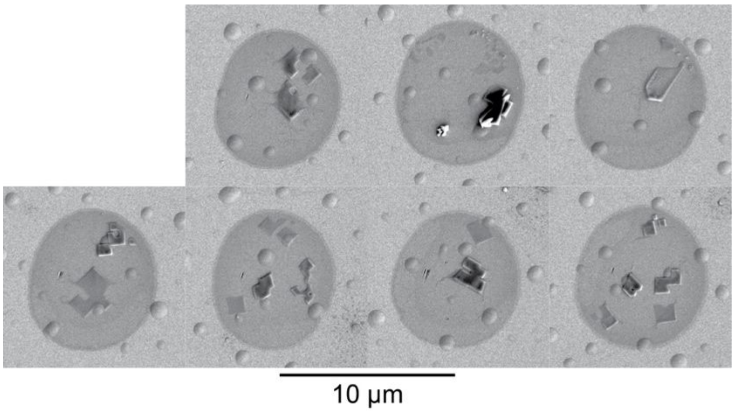


**Figure S11**. SEM images for each SECCM footprint left after the combinatorial galvanostatic experiment at 0.22 mA cm$^{-2}$ for 5 s. No lithium particles were found under these conditions. The crystals observed are from electrolyte residues.

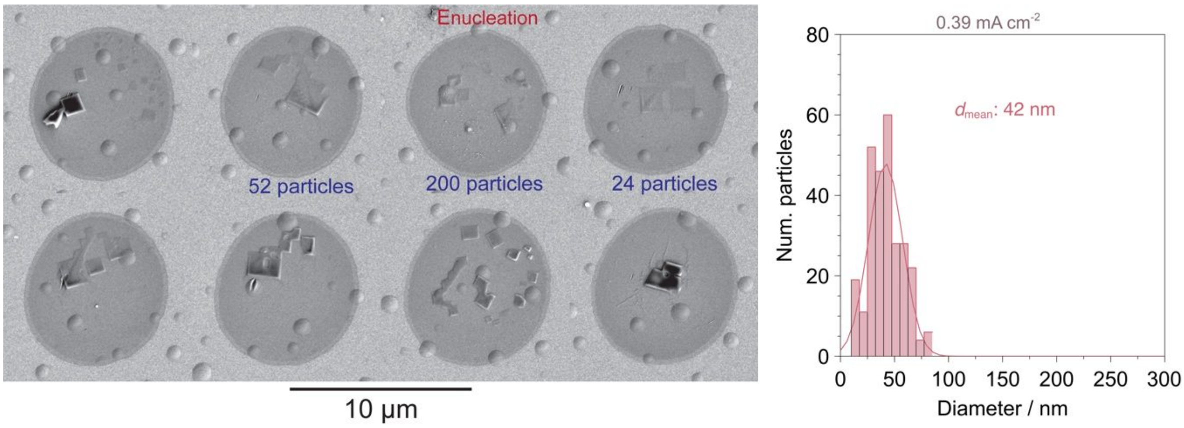


**Figure S12**. SEM images for each SECCM footprint left after the combinatorial galvanostatic experiment at 0.39 mA cm$^{-2}$ for 5 s, and the corresponding histogram of diameter distribution for the lithium nanoparticles detected. Lithium particles were only found in three repetitions, indicated in the images. The only repetition reaching the nucleation overpotential ($E_{nuc}$) is also indicated.

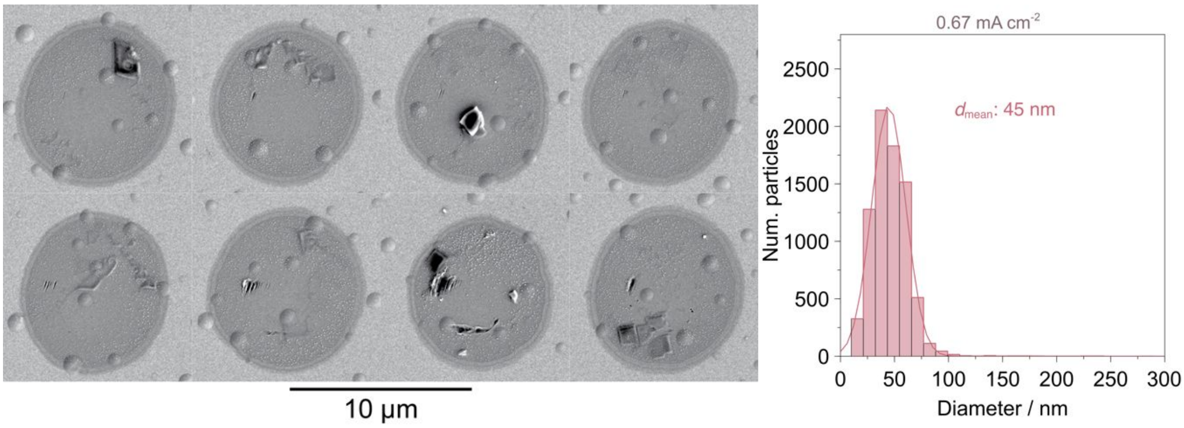


**Figure S13**. SEM images for each SECCM footprint left after the combinatorial galvanostatic experiment at 0.67 mA cm$^{-2}$ for 5 s, and the corresponding histogram of diameter distribution for the lithium nanoparticles detected.

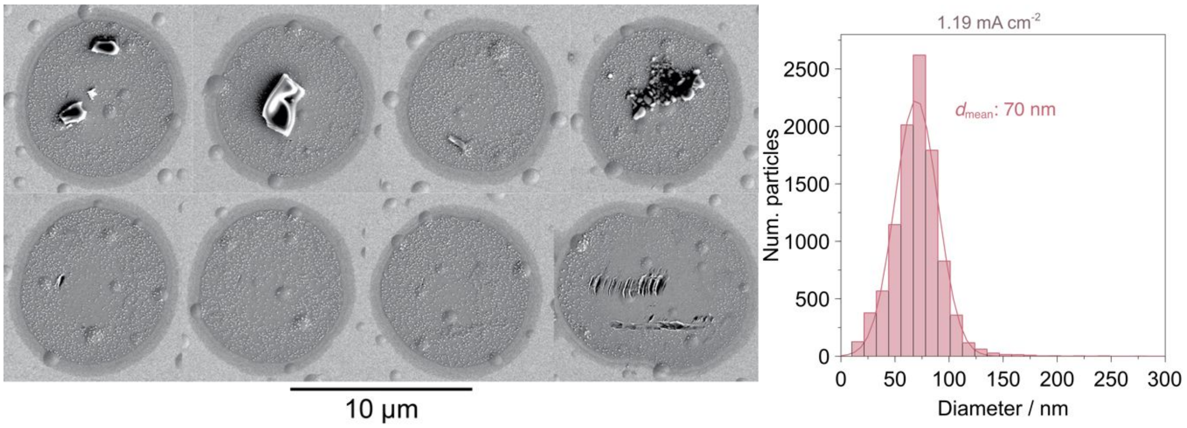


**Figure S14**. SEM images for each SECCM footprint left after the combinatorial galvanostatic experiment at 1.19 mA cm$^{-2}$ for 5 s, and the corresponding histogram of diameter distribution for the lithium nanoparticles detected.

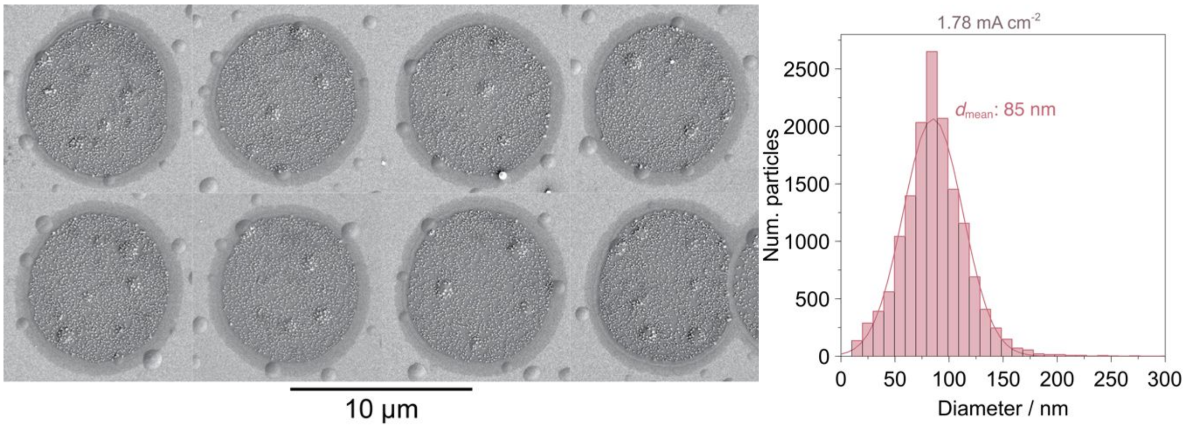


**Figure S15**. SEM images for each SECCM footprint left after the combinatorial galvanostatic experiment at 1.78 mA cm$^{-2}$ for 5 s, and the corresponding histogram of diameter distribution for the lithium nanoparticles detected.

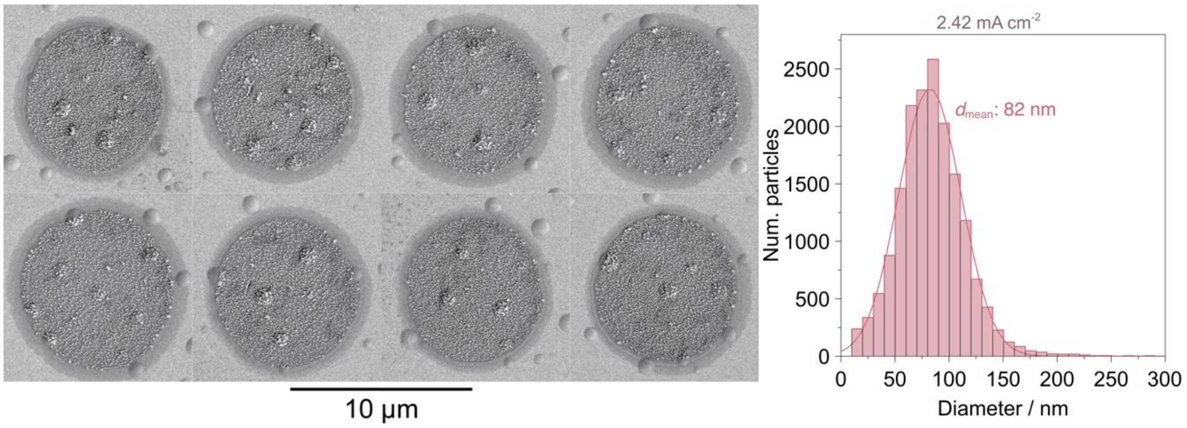


**Figure S16**. SEM images for each SECCM footprint left after the combinatorial galvanostatic experiment at 2.42 mA cm$^{-2}$ for 5 s, and the corresponding histogram of diameter distribution for the lithium nanoparticles detected.

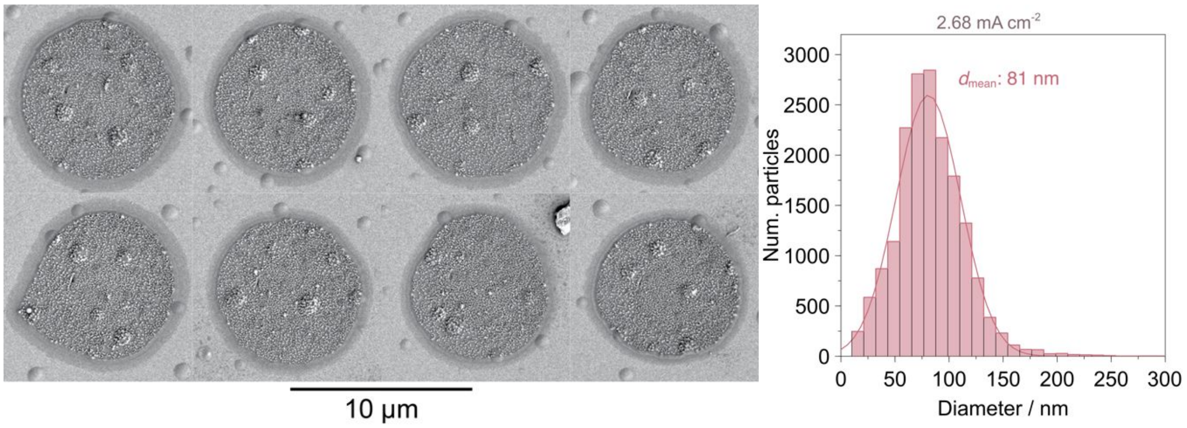


**Figure S17**. SEM images for each SECCM footprint left after the combinatorial galvanostatic experiment at 2.68 mA cm$^{-2}$ for 5 s, and the corresponding histogram of diameter distribution for the lithium nanoparticles detected.

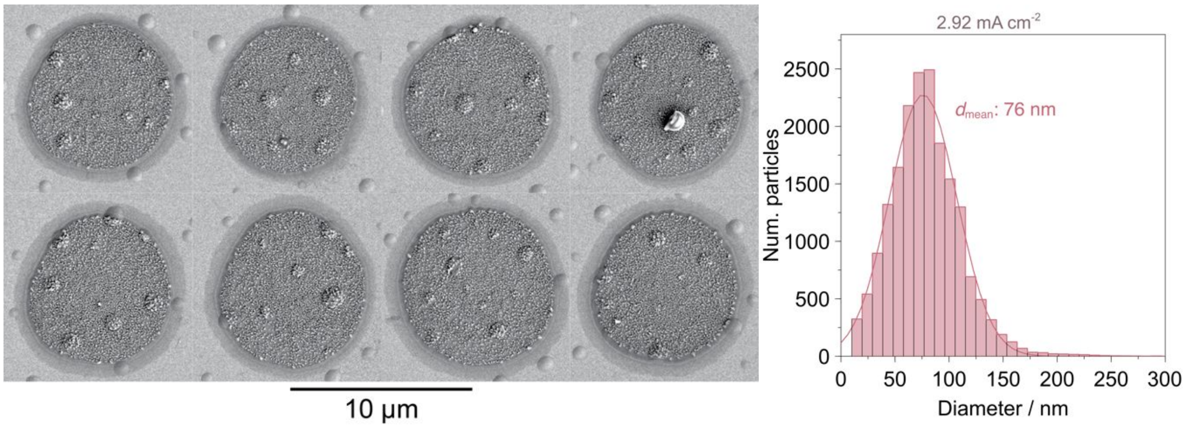


**Figure S18**. SEM images for each SECCM footprint left after the combinatorial galvanostatic experiment at 2.92 mA cm$^{-2}$ for 5 s, and the corresponding histogram of diameter distribution for the lithium nanoparticles detected.

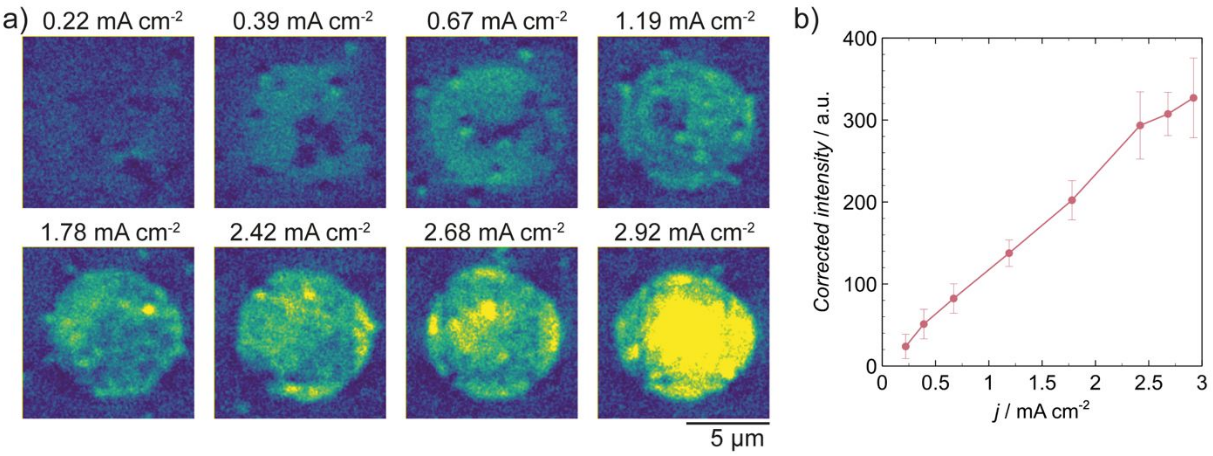


**Figure S19**. Selected IRM images for each current density recorded after the combinatorial SECCM experiment. The background intensity of each row was corrected to minimize any influence from sample tilting.

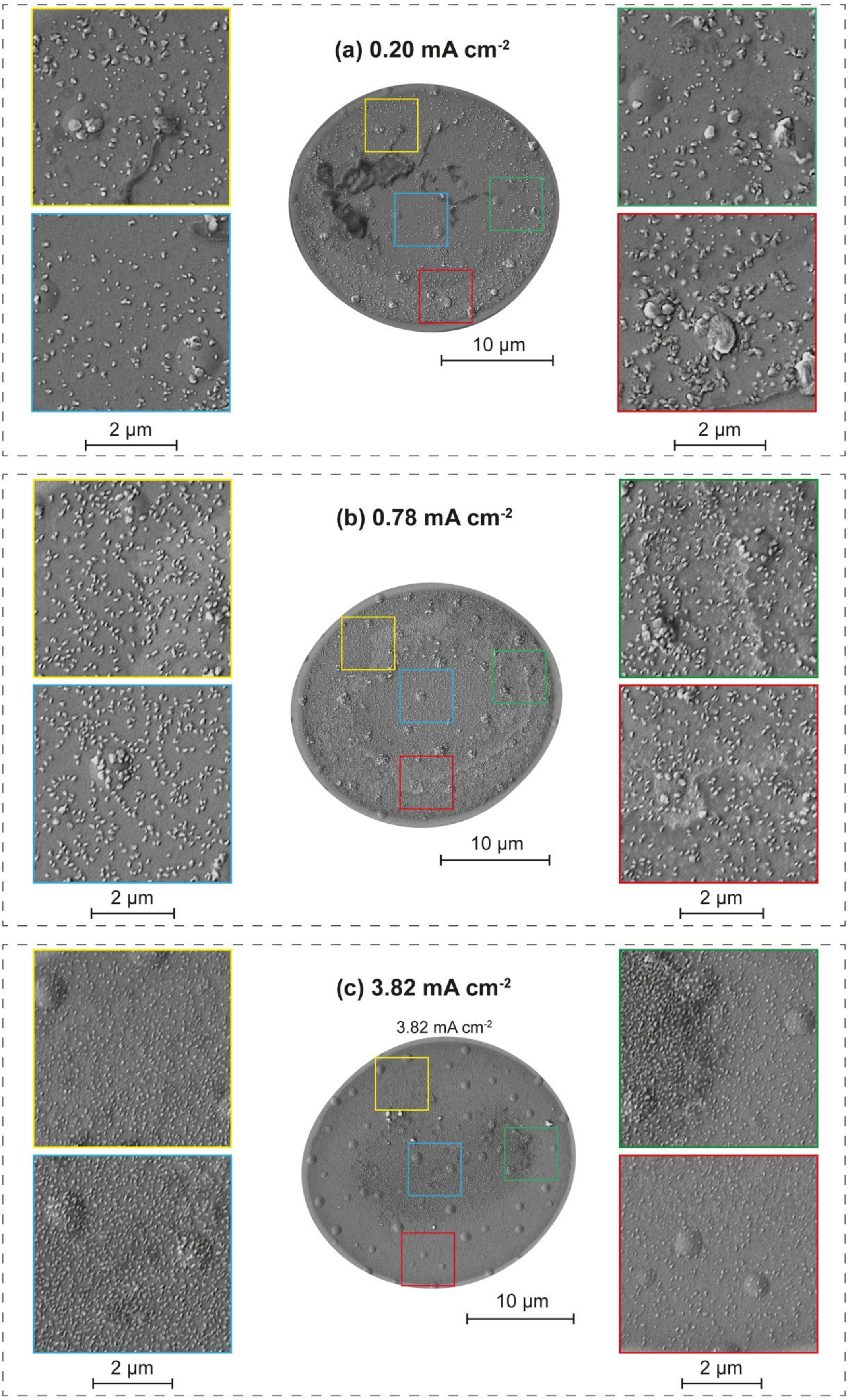


**Figure S20.** Zoomed in SEM images for SECCM galvanostatic experiments carried out at (a) 0.20 mA $cm^{-2}$ for 60 s, (b) 0.78 mA $cm^{-2}$ for 15 s, and (c) 3.82 mA $cm^{-2}$ for 3 s.

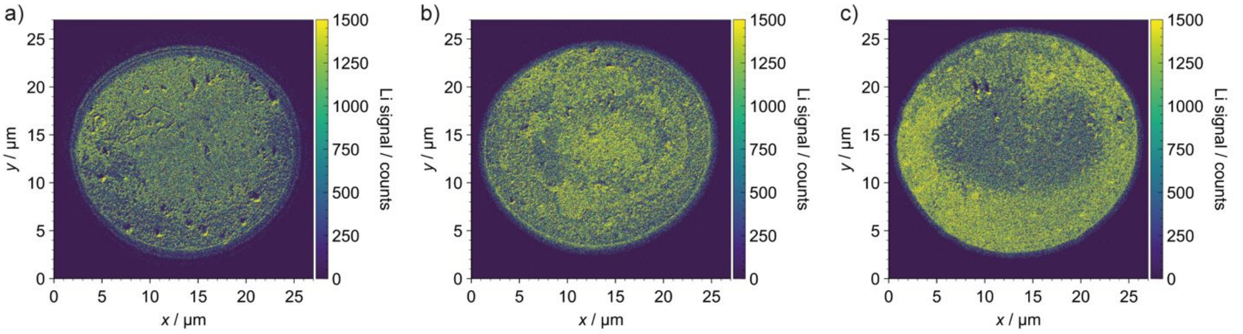


**Figure S21**. Secondary ion mass spectrometry (SIMS) $Li^{+}$ maps of the footprints left after SECCM galvanostatic experiments for 0.20, 0.78, and 3.82 mA cm$^{-2}$ for 60, 15, and 5 s, respectively. Final charge for these experiments was Q ≈ 3.2 µAh cm$^{-2}$.

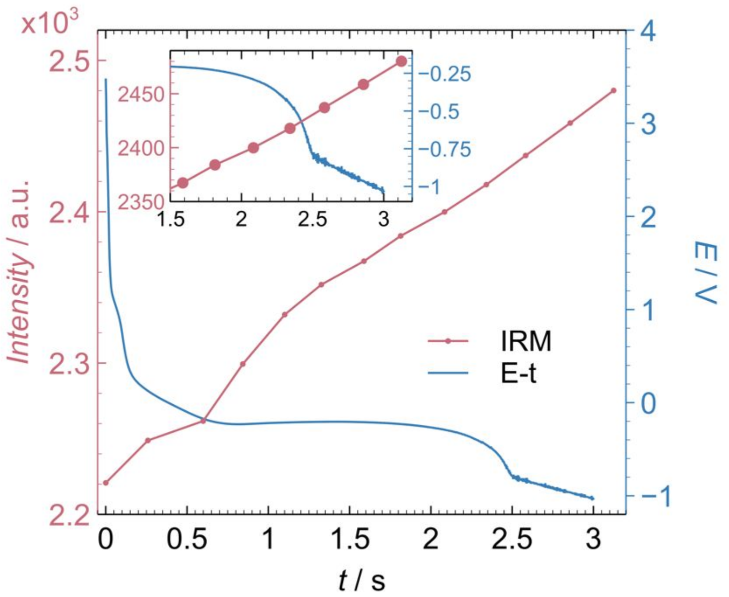


**Figure S22**. Galvanostatic *E-t* curve (blue line) and IRM intensity (red line) over the time of a SECCM galvanostatic experiment at 3.82 mA cm$^{-2}$ for 3 s. The inset illustrates the time period during which the potential shifted towards more negative values, while the rate of change in IRM intensity remained constant.

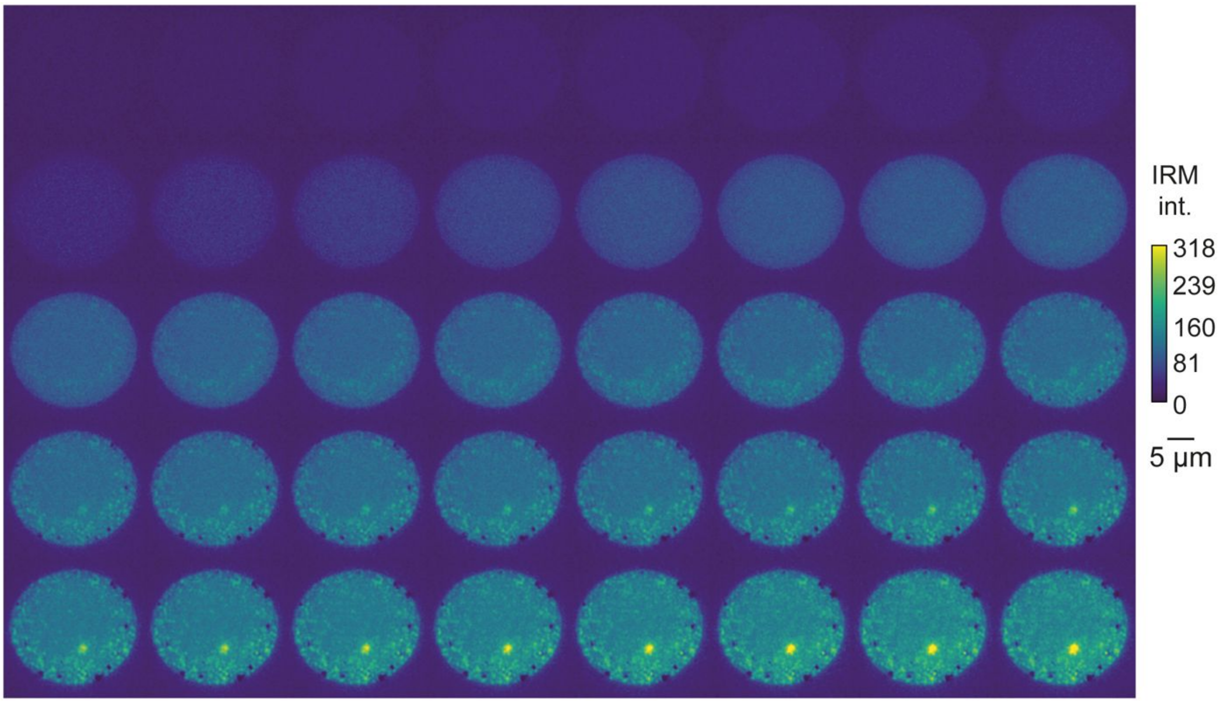


**Figure S23**. A series of frames of the IRM movie corresponding to the SECCM galvanostatic experiment at 0.20 mA cm$^{-2}$ for 60 s. One frame is shown herein for each three frames of the full movie (ca. 1.4 s). The colorbar on the right represents the IRM intensity.

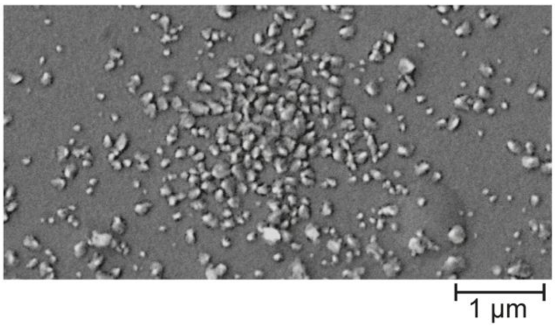


**Figure S24**. SEM image of the area for the SECCM galvanostatic experiment at 0.22 mA cm$^{-2}$ where a high density of nuclei is deposited. This hot spot is shown as a particularly bright area on the IRM images.

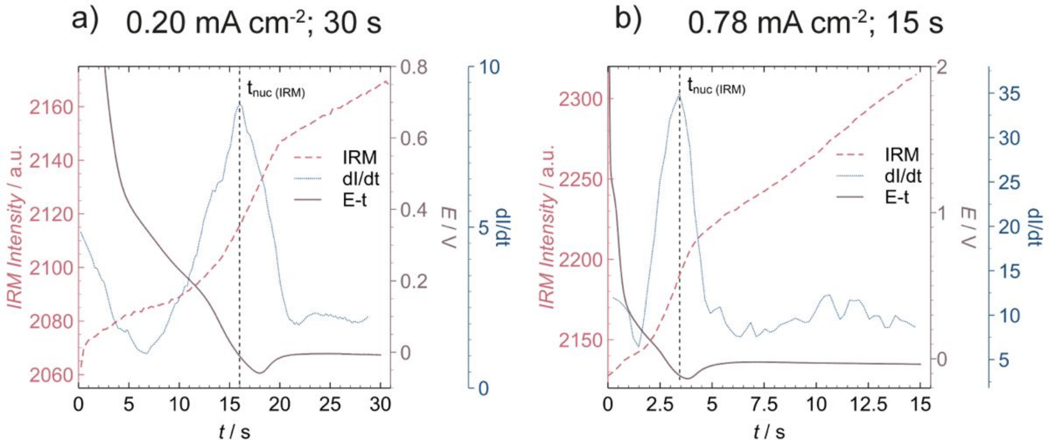


**Figure S25**. Galvanostatic *E-t* curve (brown line), IRM intensity (red line), and time derivative of the average IRM intensity (*dI*/*dt*) (blue line) over the time of SECCM galvanostatic experiments at 0.20 mA cm$^{-2}$ for 30 s (a) and 0.78 mA cm$^{-2}$ for 15 s (b). A local maximum in *dI/dt* is detected when the potential decreases towards the value where Li metal is deposited, which is termed as IRM nucleation time ($t_{nuc}$ (IRM)). This local maximum is attributed to a variation in the rate of change of the IRM intensity, resulting from the onset of the Li plating.

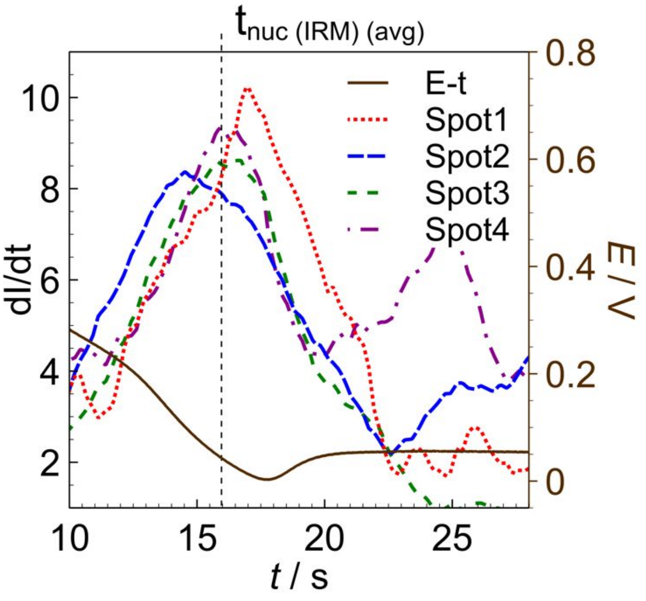


**Figure S26**. Galvanostatic *E-t* curve (solid brown line), and time derivative of the average IRM intensity (dI/dt) traces obtained from IRM analysis of selected individual LiNPs clusters shown in Figure 5c. The vertical dashed line indicates the average nucleation time from the IRM measurement, $t_{nuc(IRM)}$.

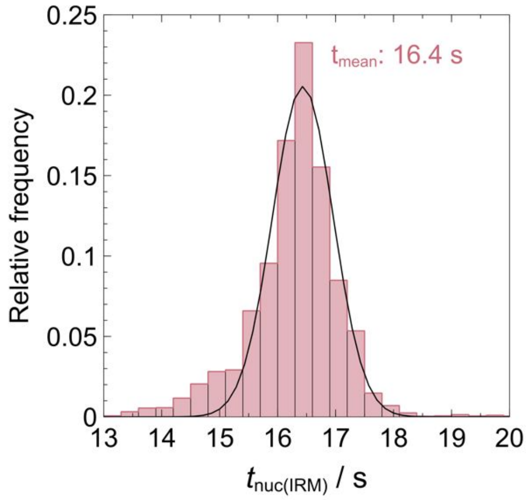


**Figure S27**. Histogram representing the distribution of all Li nucleation times obtained from IRM analysis ($t_{nuc(IRM)}$) for an experiment carried out at 0.22 mA cm$^{-2}$. The line fits to a gaussian distribution with mean of 16.4 s.

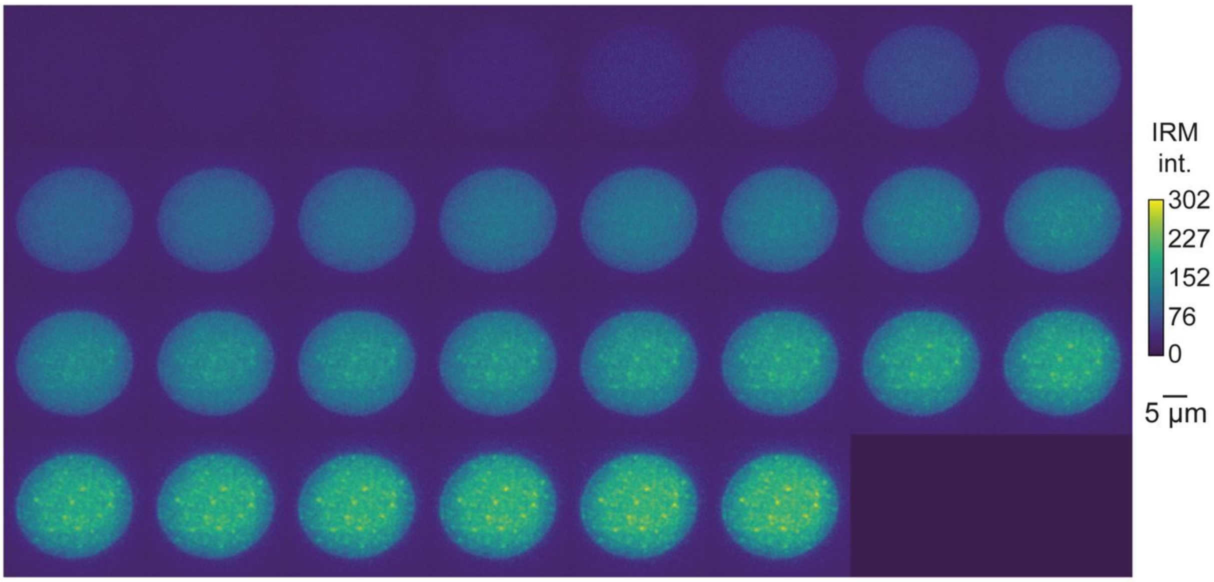


**Figure S28**. Full sequence of frames of the IRM movie corresponding to the SECCM galvanostatic experiment at 0.78 mA cm$^{-2}$ for 15 s. The colorbar on the right represents the IRM intensity.

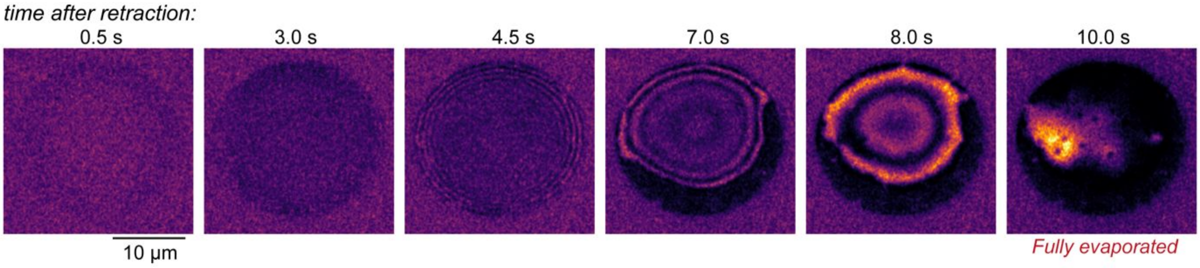


**Figure S29**. Selected frames of the IRM movie corresponding to the SECCM galvanostatic experiment at 0.20 mA cm$^{-2}$ for 60 s, after the SECCM probe has been retracted from the surface. The initial frame where probe retraction was detected served as the background frame and was subtracted from subsequent frames. This demonstrates that a droplet of $LiPF_6$/PC electrolyte can undergo evaporation in a short time.

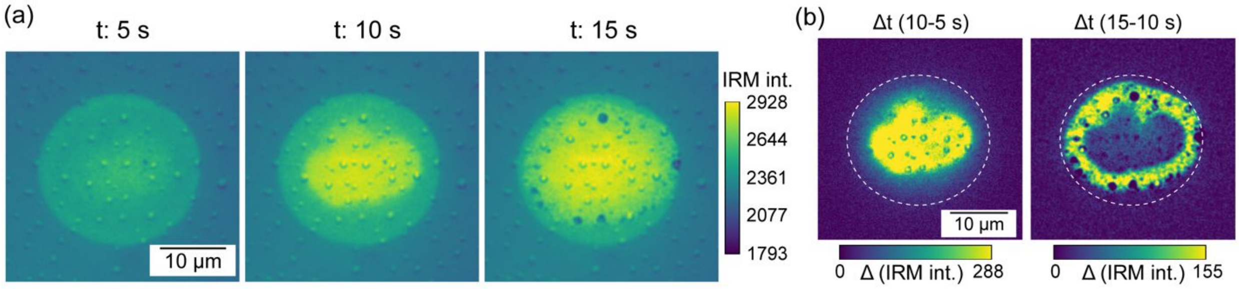


**Figure S30**. (a) Selected frames (5, 10, 15 s) of an IRM movie for a SECCM galvanostatic experiment at 3.65 mA cm$^{-2}$ for 30 s. (b) Images representing the difference in IRM intensity between the frames at 10 and 5 s and the frames at 15 and 10 s, which clearly shows the increased Li deposition at the meniscus edge at longer experimental times compared to the initial preferential deposition at the centre. Note that the appearance of a few dark blue spots in the Δt(15-10 s) frame indicates the growth of very thick Li structures due to different interference phenomena.[1,2]

## MOVIE CAPTIONS

**Movie S1**. Movie showing the IRM intensity variation during the SECCM experiment for 10 voltammetric cycles between +1.53 V and -0.21 V vs Li/Li$^+$ at a scan rate of 100 mV s$^{-1}$.

**Movie S2**. A fraction of the SECCM combinatorial galvanostatic experiment recorded by IRM imaging.

**Movie S3**. Movie showing the IRM intensity variation during the SECCM galvanostatic experiment at 0.20 mA cm$^{-2}$ for 60 s.

**Movie S4**. Movie showing the IRM intensity variation during the SECCM galvanostatic experiment at 0.78 mA cm$^{-2}$ for 15 s.

**Movie S5**. Movie showing the IRM intensity variation during the SECCM galvanostatic experiment at 3.82 mA cm$^{-2}$ for 3 s.

# REFERENCES

(1) Valavanis, D.; Ciocci, P.; Meloni, G. N.; Morris, P.; Lemineur, J.-F.; McPherson, I. J.; Kanoufi, F.; Unwin, P. R. Hybrid Scanning Electrochemical Cell Microscopy-Interference Reflection Microscopy (SECCM-IRM): Tracking Phase Formation on Surfaces in Small Volumes. *Faraday Discuss.* **2022**, *233*, 122–148.

(2) Ciocci, P.; Lemineur, J.-F.; Noël, J.-M.; Combellas, C.; Kanoufi, F. Differentiating Electrochemically Active Regions of Indium Tin Oxide Electrodes for Hydrogen Evolution and Reductive Decomposition Reactions. An in Situ Optical Microscopy Approach. *Electrochimica Acta* **2021**, *386*, 138498.
